# Preclinical and Clinical Immunotherapeutic Strategies in Epithelial Ovarian Cancer

**DOI:** 10.3390/cancers12071761

**Published:** 2020-07-02

**Authors:** Alejandra Martinez, Jean-Pierre Delord, Maha Ayyoub, Christel Devaud

**Affiliations:** 1 Cancer Research Center of Toulouse (CRCT), Institut National de la Santé Et de la Recherche Médicale (INSERM) Unité 1037, 31037 Toulouse, France; martinez.alejandra@iuct-oncopole.fr (A.M.); Delord.Jean-Pierre@iuct-oncopole.fr (J.-P.D.); maha.ayyoub@inserm.fr (M.A.); 2 Department of Surgery, Institut Claudius Regaud, Institut Universitaire du Cancer de Toulouse (IUCT), 31037 Toulouse, France; 3 Department of Medical Oncology, Institut Claudius Regaud, Institut Universitaire du Cancer de Toulouse, 31037 Toulouse, France; 4 Université Toulouse III Paul Sabatier, 31037 Toulouse, France; 5 Immune Monitoring Core Facility, Institut Claudius Regaud, Institut Universitaire du Cancer de Toulouse, 31037 Toulouse, France

**Keywords:** epithelial ovarian cancer, immune contexture, tumor microenvironment, adaptive and innate immune responses, lymphocytes, immunogenicity, myeloid cells, immunotherapy, preclinical mouse models, clinical trials

## Abstract

In the past 20 years, the immune system has increasingly been recognized as a major player in tumor cell control, leading to considerable advances in cancer treatment. While promising with regards to melanoma, renal cancer and non-small cell lung cancer, immunotherapy provides, for the time being, limited success in other cancers, including ovarian cancer, potentially due to insufficient immunogenicity or to a particularly immunosuppressive microenvironment. In this review, we provide a global description of the immune context of ovarian cancer, in particular epithelial ovarian cancer (EOC). We describe the adaptive and innate components involved in the EOC immune response, including infiltrating tumor-specific T lymphocytes, B lymphocytes, and natural killer and myeloid cells. In addition, we highlight the rationale behind the use of EOC preclinical mouse models to assess resistance to immunotherapy, and we summarize the main preclinical studies that yielded anti-EOC immunotherapeutic strategies. Finally, we focus on major published or ongoing immunotherapy clinical trials concerning EOC.

## 1. Introduction 

Ovarian cancer is the most lethal among gynecological malignancies, due to diagnosis at advanced stages of the disease and to intrinsic and acquired resistance to chemotherapy in a large proportion of patients. Although progress has been made in the treatment of ovarian cancer by more aggressive surgical approaches and the introduction of platinum-taxane regimens, the 5-year overall survival for the advanced disease is approximately 30% [1]. Small numbers of drug-resistant cells can persist and remain dormant in the peritoneal cavity, growing progressively and leading to death, despite aggressive treatment of the recurrent disease [2]. Ovarian cancer comprises remarkably heterogeneous diseases, with distinct clinico-pathological and molecular features and prognoses [3]. Despite the existence of a variety of ovarian cancer subtypes, these are treated as a single disease. The most frequent subtype, high-grade serous carcinoma (HGSC), accounts for approximately 80% of epithelial ovarian cancers (EOC). HGSC are characterized by poor prognosis and typical mutations in genes, such as *TP53* and *BRCA1/BRCA2*, that are involved in at least 96% and 22% of HGSC cases, respectively [4]. The other subtypes of EOC are less frequent and may be distinguished by other genetic alterations, such as *KRAS* mutations, occurring in 75% of mucinous carcinomas (MC), or tumor suppressor *ARID1A*, which is found to be mutated in 50% of clear cell carcinomas (CCC) [4]. Even if maintenance therapy with inhibitors of poly ADP-ribose polymerase (PARP) drove a paradigm shift in the treatment of BRCA-mutated tumors, there is still the need for new therapeutic strategies to improve outcomes in the majority of patients. 

Increasing evidence, from biological and clinical data, indicates that ovarian cancers are “immunogenic tumors” that can be recognized by the host immune system [5,6,7,8]. Indeed, EOC patients develop spontaneous antitumor immune responses, which can be detected in peripheral blood, tumors and ascites, and are likely associated with improved survival in some patients. In addition, evidence of tumor immune evasion mechanisms, associated with an immunosuppressive tumor microenvironment, and their association with shorter patient survival were also described [8]. Therapies that harness and enhance antitumor effector cells, such as immune checkpoint blockade (ICB), have led to clinical benefits for several malignancies, including melanoma, non-small cell lung cancer and renal cell carcinoma [9]. The tight relationship between preexisting antitumor immune responses and patient responses to ICB in many cancer types [10] suggests that EOC patients may benefit from immunotherapy approaches.

In this review, we will first describe the immune context of EOC, highlighting relevant major immune populations that infiltrate tumors, their related functions in immune rejection or tolerance, and their associations with clinical outcomes. We will then discuss the immunotherapeutic strategies that were investigated in EOC mouse models, for their potential to prevail over the highly immunosuppressive EOC tumor microenvironment, including ICB, adoptive T-cell therapy (ACT), immunosuppressive microenvironment targeting, cytokine-based therapy and cancer vaccines. Finally, we will present an overview of clinical trials designed to assess the potential benefit of immune intervention in the treatment of EOC patients.

## 2. Immune Responses in Ovarian Cancer

### 2.1. Importance of the Immune Contexture in Cancer 

**Figure 1:**
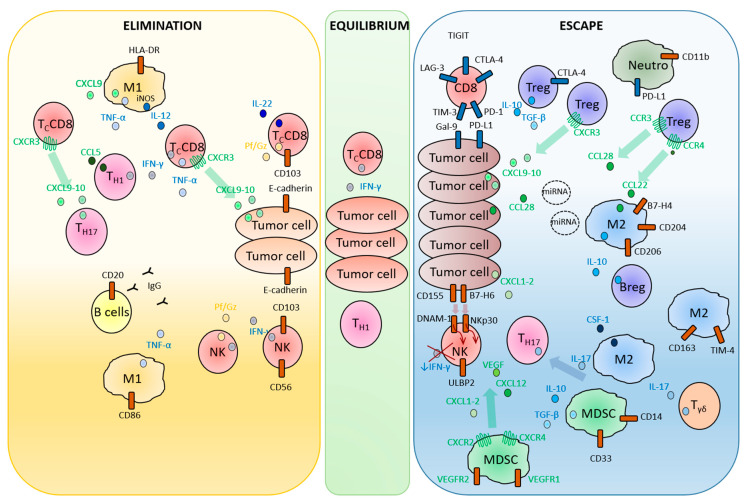
The immune landscape of epithelial ovarian cancer (EOC): cells, mechanisms and factors involved in tumor elimination or escape. EOC tumor elimination implicates cytotoxic cells, such as cytotoxic CD8^+^ T-cells (T_C_CD8) or natural killer (NK) cells, that produce cytolytic perforin/granzymes (Pf/Gz), and various effector cytokines and CD20^+^ B cells, as well as IgG-producing plasmocytes. T_C_CD8 are recruited to the tumor site via chemokine receptor expression and chemokines released in the microenvironment by other cells, including T_H_17. Both T_C_CD8 and CD56^+^ NK cells are retained at the tumor site through CD103–E-cadherin interactions. Finally, myeloid cells such as CD86^+^HLA-DR^+^ type-1 polarized macrophages (M1) perform antitumor roles through iNOS enzyme expression, secretion of TNF-α and T_H_1-T_C_CD8-stimulating cytokine IL-12. The intermediate equilibrium phase involves adaptive immunity components, in particular INF-γ^+^ TcCD8 and Th1 cells. In contrast, cells such as regulatory CD4^+^ T-cells (Treg), type-2 macrophages (M2), B regulatory cells (Breg), myeloid-derived suppressor cells (MDSC), suppressive neutrophils (Neutro), IL-17-producing γδ T-cells (Tγδ) and T_H_17 generate an immunosuppressed microenvironment, favoring tumor escape. Treg are recruited to tumors via CXCL9, CXCL10, CCL28 or M2-secreted CCL22, and prevent antitumor response through IL-10, TGF-β and cell–cell contact-dependent mechanisms. In addition, M2 produce miRNA-containing exosome, IL-10 and CSF-1, all of which promote tumor progression. MDSC are recruited to tumors through vascular endothelial growth factor receptor (VEGFR)1, VEGFR2, CXCR2 and CXCR4 expression, and VEGF, CXCL1, CXCL2 (CXCL1-2) and CXCL12 in the microenvironment. Breg and MDSC also secrete immunosuppressive IL-10 and TGF-β. Their recruitment, together with M2, is increased through T_H_17-secreted IL-17. Finally, T_C_CD8 and NK cells’ antitumor functions are inhibited via checkpoint expression, and via downregulation of DNAM-1 and NKp30, following binding with tumor cells-expressed CD155 and B7-H6, thus triggering downregulation of IFN-γ production.

Tumors are complex networks, in which tumor cells are surrounded by an intricate cellular microenvironment that encompasses immune cells. A large numbers and types of immune cells have opposite effects, leading to either the hinderance or promotion of tumor development [11]. Thus the immune contexture, defined by density, composition and functional state of the immune tumor-infiltrate, is a determining factor of tumor progression, and predictive for a patient’s prognosis and response to treatment [10,12], as recently validated for EOC [13]. Immune contexture is variable among tumor histological types and across anatomical sites in which tumors grow, thus considerably contributing to the tumor type- and localization-dependent outcomes [14,15]. We and others have established that, in addition to tumor cell-intrinsic factors, tumor growth regulation depends on local cues driven by tissue environment-specific components [15,16,17,18]. In agreement with this concept, distinct immune microenvironments were found in multiple tumor sites from a single EOC patient [7], or within a single organ [19], possibly explaining the heterogeneous fates of metastatic lesions. In a case of HGSC, immunogenomic approaches demonstrated that regressing and stable metastatic lesions were infiltrated by tumor-specific T lymphocytes, while, concomitantly, progressing metastases were “immune excluded”, and characterized by poor immune cell infiltration [7]. Tumors are infiltrated by adaptive immune cells (naïve, memory, effector, regulatory CD4^+^ or CD8^+^ T lymphocytes, and B cells) and innate immune cells [natural killer (NK) cells, macrophages, myeloid derived suppressor cells (MDSC), dendritic cells (DC), neutrophils and mast cells] [20] (Figure 1). In cancer, including EOC, each of these cellular components can paradoxically constrain and promote tumor development through a three-phase process referred to as cancer immunoediting, occurring during tumor progression and also in patients receiving anticancer immunotherapies [21]. In the first phase, called “elimination”, immune cells can recognize and kill recently transformed malignant cells. During the second “equilibrium” phase, the rare tumor variants that have survived elimination can enter a non-growing dormant state that can last for long periods of time, during which immunogenicity is edited. Finally, in the third “escape” phase, tumor cells exit dormancy and proliferate again with the help of the immunosuppressive microenvironment [21] (Figure 1). The cancer immunoediting process provides the fundamental basis for studying immunity to EOC, and for the rational design of immunotherapies against EOC.

### 2.2. Adaptive Immune Responses in EOC 

T lymphocytes: These represent central components of antitumor immunity. In EOC, CD8 and CD4 T-cells infiltrate tumor tissues and ascites, and exert antitumor functions through specific recognition of tumor antigens (TA) [22]. Following seminal studies on the key role of tumor-infiltrating lymphocytes (TIL) in predicting patient outcome [5,12,23], an important body of data corroborates the correlation between TIL and favorable prognosis in multiple solid cancers, including EOC. The survival benefit of TIL in EOC was actually studied as early as 1991 [24]. Coukos and colleagues, in particular, observed that 55% of advanced-stage EOC patients, with detectable intraepithelial CD3^+^ TIL, had a 5-year survival of 38%, in contrast to 4.5% in patients whose tumors contained no TIL [5].

CD8^+^ T lymphocytes: Although the overall CD3^+^ TIL population was shown to provide survival benefits for EOC patients [5], among CD3^+^ cells, intraepithelial CD8^+^ TIL appear to be central players in tumor immune control [25,26,27]. A meta-analysis, comprising 1815 ovarian cancer patients, encompassing all tumor grades, stages and histologic subtypes, validated CD8^+^ TIL as a robust outcome predictor [6]. In HGSC, CD8^+^ T-cells’ migratory abilities, through the CXCL9/CXCL10-CXCR3 axis [28], as well as their tissue residency phenotype, characterized by α_E_ integrin CD103 expression [29,30], were associated with increased survival. St Paul et al. demonstrated that IL-22, produced by highly cytotoxic CD8^+^ TIL, was correlated with improved recurrence-free survival in EOC patients [31]. Following tumor infiltration, CD8^+^ TIL performs antitumor effector functions, through the exocytosis of perforin and cytotoxic granzymes, or through the release of interferon (IFN)-γ and tumor necrosis factor (TNF)-α [32]. In their seminal study, Coukos and colleagues highlighted that intratumoral TIL were associated with increased intratumor expression of IFN-γ and IL-2 [5]. As tumors progress, TIL become gradually exhausted, with increased expression of inhibitory receptors and reduced effector functions [33]. In EOC, CD8^+^ TIL express immune checkpoints (IC), including programmed cell death 1 (PD-1), cytotoxic T-lymphocyte antigen-4 (CTLA-4), T-cell immunoreceptor with Ig and ITIM domains (TIGIT), T-cell immunoglobulin and mucin domain-containing (TIM)-3 [34], the lymphocyte-activation gene (LAG)-3 [35] and CD112R (PVRIG) [36]. We and others have demonstrated in EOC that, despite their exhausted status, CD8^+^ TIL exhibit a sustained potential for cytokine production and proliferation [34,37]. 

CD4^+^ T helper lymphocytes: CD4^+^ T helper (T_H_) cells provide support for CD8^+^ T-cell proliferation and expansion, via activation of antigen-presenting cells and the secretion of cytokines such as IFN-γ [38]. Similar to CD8^+^ TIL, a high frequency of CD4^+^ TIL correlates with improved EOC patient survival [39,40,41,42]. We previously demonstrated that the circulating NY-ESO-1-specific CD4^+^ T-cells in EOC patients are most commonly IFN-γ-secreting T_H_1, and not immunosuppressive FOXP3^+^ T regulatory cells (Treg) [43]. In addition, we developed MHC Class II/NY-ESO-1 tetramers [44], allowing direct ex vivo quantification of NY-ESO-1-specific CD4^+^ T-cells in EOC ascites and solid tumor masses [45]. We showed that T_H_1 cells accumulating in ovarian tumors are able to maintain an antitumor effector phenotype, despite a concomitant high infiltration by FOXP3^+^ Treg [45]. In an EOC preclinical model, T_H_1 cells produced high levels of CCL5, enabling the recruitment and activation of DC in the tumor microenvironment, which eventually induces tumor-specific CD8^+^ T-cell activation [46]. In addition, T_H_17 cells, accumulating in EOC tumor ascites, are able to recruit CD8^+^ effector T-cells through the production of CXCL9 and CXCL10, thus contributing to antitumor immunity [47]. Accordingly, T_H_17 are associated with improved patient survival [47]. In contrast, another study demonstrated that, in mice and patients, chronic production of TNF-α in ovarian tumors promotes IL-17 production by T_H_17, leading to myeloid cell recruitment, which in turn participate in tumor progression [48]. Since then, other studies have confirmed the accumulation of T_H_17 in EOC, although their correlation with patient survival remains debatable [49,50]. 

CD4^+^ T regulatory lymphocytes (Treg): CD4^+^FOXP3^+^CD25^+^ Treg are strong suppressors of antitumor immunity through mechanisms including cell–cell interactions, via, for instance, CTLA-4 expression, and transforming-growth factor (TGF)-β and IL-10 cytokine secretion [51]. In EOC tumors, Treg preferentially accumulate in tumors and ascites [52,53]. Treg infiltration in ovarian tumors and ascites often correlates with poor patient outcome [54,55], as they specifically suppress antitumor T-cells in vivo, and contribute to tumor growth [56]. Treg infiltration in ovarian tumors increases throughout EOC progression. Fiavolá et al. characterized a strong T_H_17 response in the early-stage disease, while stage II tumors are infiltrated by high numbers of Treg, as well as macrophages and DC, which produced CCL22, enabling the further recruitment of Treg [57]. Other chemokines induce Treg recruitment in ovarian tumors, such as CCL28, the overexpression of which was associated with the poor outcome in EOC patients [58]. In addition, we previously demonstrated that a major subset of CXCR3^+^T-bet^+^Helios^+^FOXP3^+^ Treg selectively accumulated in ovarian tumors, and suppressed the proliferation of IFN-γ production by effector T-cells [59]. In contrast, Treg frequencies may also represent significant predictors of favorable prognosis in patients with familial ovarian cancer [60], or in optimally debulked HGSC patients. Nonetheless, the improved survival was also positively associated with other TIL markers, such as CD8 and CD3 [61]. 

Antigen-specific T-cell responses in EOC: Tumor antigens (Ag), recognized by αβ T lymphocytes, can be classified into multiple categories, including neoantigens that are derived, specifically in tumors, from genetic alterations, cancer-testis Ag (CTA), tissue differentiation Ag, overexpressed Ag, and other factors [62]. Importantly, the good prognostic value of tumor Ag is related to the appropriate expression of Ag-processing machinery, as well as to a strong T-cell infiltration of ovarian tumors [63]. The neoantigen load in EOC is rather low, as comprehensive genomic profiling revealed a low overall tumor mutational burden among subtypes (e.g., 3.6 mutations/megabase in HGSC) [64]. In addition, like in other tumor types, few mutations (only 1.3%) are recognized by autologous tumor-associated T-cells in HGSC patients [65]. Nonetheless, comprehensive analyses of advanced EOC-associated T-cells revealed that, despite a relatively low number of somatic mutations, the identification of neoepitope-specific CD8^+^ T-cells is achievable in EOC [66]. The presence of neoantigen-reactive T-cells in EOC patients was associated with improved survival [31]. CTA have been extensively studied in EOC, in particular the frequently expressed and highly immunogenic Ag NY-ESO-1. We previously delineated the phenotypes of NY-ESO-1-reactive CD4^+^ T_H_1 cells [43,45] and CD8+ cytotoxic T-cells [34] accumulating in ovarian tumors. In particular, we showed that NY-ESO-1-specific CD8^+^ EOC TIL are characterized by high IC expression, as well as by the expression of CD39 and tissue-resident memory T-cell markers [34]. Evidence of spontaneous humoral and cellular immune responses to NY-ESO-1 was reported in EOC patients [67,68], supporting the strategy of targeting it with vaccination [69]. In addition, recent mass spectrometry analyses in EOC tumors confirmed the presentation of peptides derived from other CTA, such as CAGE, which could be recognized by antigen-specific TIL [70], and MUC16, which are presented by MHC-I molecules and are highly immunogenic in vitro [71]. 

B lymphocytes: A body of data supports the role of B cells and plasma cells in shaping cancer immune responses [72]. In EOC, stromal or intraepithelial B lymphocytes are detected in tumors, but their role in tumor progression is debated [61]. Indeed, several B-cell subtypes, including naïve and memory B cells and regulatory B cells (Breg), accumulate in EOC and exhibit both pro- and antitumor functions [73]. Combined CD20^+^ B and CD8^+^ T HGSC TIL shows a higher prognostic value than CD8^+^ TIL alone [26,74]. In addition, Balkwill’s group demonstrated that HGSC omental metastases are infiltrated by memory B cells, directed at a restricted repertoire of Ag and producing tumor-specific IgG, thus supporting the development of an antitumor response [75]. Conversely, studies demonstrated that B cells infiltrating epithelial tumor tissues and omental metastases negatively correlate with patient survival [76,77,78]. Indeed, Breg subtypes, able to produce IL-10 and suppress the antitumor immune response, have been described in many cancer types, including EOC [79]. In particular, IL-21 was shown to induce granzyme B (GrB)-expressing Breg, which may reside within the EOC microenvironment and contribute to the suppression of adaptive immune responses by Treg-like mechanism [80].

### 2.3. Innate Immunity in the Context of EOC 

Natural killer (NK) cells: Through an equilibrium between signals transduced by inhibitory and activating receptors, these innate lymphoid cells are able to exert immediate cytolytic activity through lytic granule release [81]. Increased numbers of CD56^+^ NK cells were reported in HGSC and endometrioid carcinoma (EC) subtypes, compared to MC and CCC [40], in particular in ascites. However, they seem to be functionally impaired [82,83]. Cells in the EOC microenvironment can express B7-H6 and CD155 that, respectively, trigger the downregulation of the NK cell-activating receptors NKp30 and DNAM-1, hence impairing their IFN-γ production and cytolytic functions [84,85]. In addition, the MUC16 Ag, expressed by EOC cells, may protect them from recognition by NK cells, by inhibiting synapse formation, leading to the increased ability of EOC cells to metastasize [86]. Thus, the prognostic value of EOC-infiltrating NK cells is still under debate. Expression of NK cell receptor ligand ULBP2 by EOC tumors was correlated with less infiltration of T-cells, and poor prognosis [87]. In contrast, NK cell co-infiltration with cytotoxic T-cells into tumors has been associated with better EOC patient survival [29,78]. CD103-expressing NK cell infiltration, along with T-cell infiltration, correlates with increased 5-year survival rates in EOC patients [88]. The presence of CD56^+^ NK cells in EOC ascites was also associated with better patient outcome, and their antitumor functions could be boosted by IL-15-receptor stimulation [89]. Increased NK cell activity was also detected in the peripheral blood of EOC patients, and was correlated with significantly increased progression-free survival [90]. The ability of NK cells to mediate the killing of EOC tumors has been extensively described [91]. For instance, NK cells efficiently eliminate EOC tumor cells in 3D tumor spheroids, and reduce tumor progression in EOC tumor-bearing mice [92]. 

Macrophages: These myeloid cells can typically exert dual roles in cancer, by enhancing antitumor immune responses, or supporting progression through the establishment of an immunosuppressive microenvironment [93]. In EOC, long-lived resident macrophages and short-lived infiltrating macrophages are the two main sources of macrophages, both acquiring specific phenotypes through signals from the surrounding microenvironment [94]. In EOC, both resident and infiltrating macrophages differentiate into a mixed population of pro-tumorigenic type 2-like macrophages (M2) and antitumor type 1-like macrophages (M1), and constitute, in ascites in particular, a large fraction of immune cells [95]. Reinartz et al. analyzed the transcriptome of human ovarian tumor-associated macrophages (TAM), and revealed mixed populations, expressing either CD163 and IL-10 M2 markers or CD86 and TNF-α M1 markers. In this study, CD163 surface expression was correlated with ascites IL-6 and IL-10 concentrations, and was inversely correlated with patient relapse-free survival [96]. In EOC tumors and ascites, TAM are characterized by the expression of M2-related markers, such as CD163, CD204, CD206 and IL-10 [97], and their presence correlates with tumor progression [60,98] and poor patient survival [99]. Functionally, EOC TAM suppress antitumor responses by producing Treg-attracting chemokines, such as CCL22 [56] or CCL18 [100], and by expressing co-inhibitory molecules, such as B7-H4, which inhibits T-cell cytotoxicity [101]. Zhou et al. demonstrated the ability of EOC TAM to release miRNAs-enriched exosomes, which suppress the STAT3 transcription factor and induce a Treg/T_H_17 imbalance, thus facilitating EOC progression and metastasis [102]. In addition, TAM secrete the colony-stimulating factor 1 (CSF-1) that contributes to tumor growth, invasion and metastasis in serous and mucinous EOC [98]. EOC TAM, such as B7-H4^+^ TAM, are also able to interact with other immunoregulatory cells such as Treg, which in turn stimulate IL-10 and IL-6 production by TAM, further enhancing the immunosuppressive tumor microenvironment and contributing to poor patient outcome [54]. Protumorigenic TAM polarization and its functions in EOC are regulated by transcription factors such as proliferator-activated receptor (PPAR) β/δ, targeting multiple genes, such as *LRP5* or *CD300A*, associated with tumor progression [103], or GATA3, the expression of which was associated with poor prognosis in HGSC [104]. While TAM are key suppressors of antitumor immune responses, they also increasingly appear to be crucial in promoting the development of the premetastatic niche and metastatic spreading during EOC [105,106,107], in particular a unique subset of CD163^+^Tim4^+^ resident omental macrophages [108]. 

In contrast, some studies highlighted that intratumor, but not stromal, M1-polarization, characterized by HLA-DR and inducible nitric oxide synthase (iNOS) expression, may also be associated with the extended overall survival of EOC patients [109,110]. In EOC ascites, M1 are able to produce IL-12 and TNF-α, which will prompt a cytotoxic T-cell response against tumor cells [111,112]. Recently, Coukos and colleagues demonstrated that IFN-γ induces CXCL9-expression by macrophages and DC, thus orchestrating T-cell infiltration in EOC tumors in patients responsive to immunotherapy [113].

Myeloid Derived Suppressive Cells (MDSC): These are a heterogeneous population of immature myeloid cells, which contribute to tumor progression and potently dampen antitumor immune responses, through mechanisms including the expression of immunosuppressive mediators (e.g., arginase, TGF-β, IL-10) [114]. Cui et al. established, for the first time, the clinical impact of CD33^+^ MDSC, which were significantly associated with shorter overall survival and a reduced disease-free interval in HGSC [115]. Recently, the same group determined that the monocytic-MDSC subset appears to be the best predictor of poor survival [116]. MDSC, in particular CD14^+^HLA-DR^-/lo^ MDSC, are enriched in peripheral blood and ascites during EOC, and their suppressive activity is correlated with ascites-derived IL-6 and IL-10 [117]. The immune profiling of EOC patient blood demonstrated a major involvement of MDSC in innate immunosuppression [118]. MDSC recruitment induces EOC progression, and may involve chemokine receptors such as CXCR2, which ligands are upregulated in tumor cells via *Snai1* transcriptional factor action [119]. In addition, prostaglandin (PG) E2 appears to play a central role in CXCL12 production and CXCR4-mediated MDSC accumulation, as demonstrated by Obermajer et al., who correlated the PGE2 and CXCL12 levels in EOC ascites with the presence of CD11b^+^CD14^+^CD33^+^CXCR4^+^ MDSC [120]. Vascular endothelial growth factor receptor (VEGFR) expression in EOC tumors can induce MDSC recruitment and inhibit local immunity [121]. However, while targeting VEGF to inhibit MDSC recruitment seems an interesting therapeutic option, it may also trigger, in parallel, tumor hypoxia and GM-CSF expression, which will sustain MDSC recruitment in ovarian tumors [122].

Other cells: γδ T-cells, in particular the Vδ1^+^ subtype, were significantly increased in EOC patient tumors compared to normal ovarian tissue [123]. Rei et al. demonstrated, using a syngeneic EOC mouse model, that Vγ6^+^ γδ T-cells were able to promote tumor growth through secretion of IL-17, allowing the recruitment of suppressive peritoneal macrophages [124]. Abundant IL-17-producing γδ T-cells are positively correlated with larger tumor sizes and lymph node metastases in advanced EOC patients [125]. In addition, neutrophils are contributors to innate immunity, representing new biomarkers of EOC outcome and new therapeutic targets [126]. Indeed, a high neutrophil-to-lymphocyte ratio is predictive of poor overall survival in advanced stage EOC [127]. Neutrophil influx into the omentum was identified, in orthotopic mouse EOC models, as a prerequisite premetastatic step through the formation of neutrophil extracellular traps [128]. Neutrophils, exhibiting a suppressor phenotype, can also suppress T-cell antitumor activity in the EOC microenvironment [129], for instance through upregulation of PD-L1 [130].

## 3. Preclinical Investigations for the Development of Effective Immunotherapies in Ovarian Cancer

### 3.1. Use of Mouse Models for the Design of Immunotherapies

Mouse models have permitted considerable advances in the understanding of EOC biology and the development of therapeutic strategies, including immunotherapy. They have been shown to recapitulate the anatomical features of various human EOC subtypes, mimicking tumor growth, metastatic spread and the tumor immune microenvironment, and recapitulating patient responses to therapies [131]. Important characteristics, relevant to most human EOC subtypes, have been taken into account in the design of EOC mouse models. Genetic modifications, for instance, are well recapitulated in genetically engineered mouse models (GEMM), including genetic alterations in *Tp53* and *Brca* genes [132]. GEMM are relevant models for assessing immunotherapy efficacy, as genetic alterations, such as those occurring in the *Tp53* gene, may be involved in modulating the tumor immune microenvironment, such as in the increased expression of PD-L1 [133] or the production of pro-inflammatory cytokines [134]. Recently, Balkwill and colleagues used GEMM, a knockout for *Tp53*, *Brca2* and *Pten* genes, to establish new syngeneic EOC mouse cell lines [135]. Once implanted orthotopically, the tumors develop microenvironments relevant to human primary EOC tumors and metastases, thus opening new windows for studying immunotherapy in EOC preclinical models [135].

The location of transplanted tumors in EOC mouse models is key, as the immune microenvironment’s composition is dependent on the tumor’s anatomical location [16]. While mice injected subcutaneously (SC) develop tumors readily accessible for the evaluation of the response to treatment, these tumors do not constitute an immune microenvironment representative of the human disease [16]. In comparison to SC mouse models, EOC orthotopic mouse models, achieved by surgically implanting tumors in the bursa ovari (mouse counterpart of human ovary) or by injecting tumors intraperitoneally (IP), mimic human tumor histology, vasculature, metastatic biology and immune microenvironment formation [136]. Mice with deficient immunity are used for the implantation of human tumor cell lines, most frequently SK-OV-3 and A2780 cells, or patient-derived xenografts (PDX) directly harvested from EOC patients. However, these humanized mouse models lack the appropriate immune microenvironment when human tumor cells are xenografted, and probably maintain not much time the patient’s immune tumor microenvironment following PDX transplantation, hence limiting their usefulness in the evaluation of immunotherapy. Nevertheless, they can by valuable in testing human adoptive T-cell therapy, or when human tumor cells are co-implanted with human immune components. For instance, an HGSC-PDX model was validated to assess the efficacy of anti-PD-1 therapy, by adoptively transferring in vitro-expanded autologous TIL into these mice [137]. Created syngeneic EOC mouse models, particularly using the ID8 cell line, established in 2000 by Roby and colleagues [138], is probably the most commonly employed method in the development of immunotherapies such as ICB [139,140] or the DC vaccines [141]. The peritoneal tumors generated by IP injection of ID8 cells (a model widely described by the Balkwill’s group) develop a complex microenvironment, with SMA^+^ fibroblasts, CD3^+^ T-cells, CD68^+^ macrophages and neo-vasculature [142]. However, the ID8 model does not contain most of the EOC-associated mutations [143], including *Tp53* and *Brca2* mutations, which may be artificially introduced into the cells using CRISPR-Cas9 technology [144].

### 3.2. Preclinical Assessment of Immunotherapies

Immune Checkpoint Blocade: IC expressed on T-cells either enhances or suppresses T-cell activation following binding to their ligands. ICB, using monoclonal antibodies (mAb), is currently considered as the most effective immunotherapy in many cancers, with most studies assessing the inhibitory IC PD-1 or its ligand PD-L1, and CTLA-4 [145]. PD-L1 expression in ovarian cancer cells, in particular the widely used ID8 cell line, was demonstrated to repress T-cell antitumor response [146]. Used as monotherapy, neither ICB (anti-PD-1 and anti-CTLA-4) nor activating antibodies (anti-OX40 and anti-CD137) had any significant impact on ID8 tumor-bearing mouse survival [139,140], possibly due to the compensatory upregulation of additional checkpoints on T-cells, such as LAG-3 [147]. Combination ICB therapies, however, proved efficient in EOC preclinical models. When the PD1/PD-L1 pathway blockade is combined with stimulatory anti-OX40 [140], or with anti-CTLA-4 and anti-CD137 [139,148], ID8-tumor bearing mice achieve a prolonged survival, and exhibit an increased CD8^+^ and Foxp3^-^CD4^+^ T-cell-to-Treg ratio, as well as a reduction of MDSC. Similarly, combined anti-PD-1 blockade and glucocorticoid-induced TNFR related protein (GITR)-stimulating mAb induces potent antitumor immunity in the ID8 EOC mouse model, which can be further promoted by chemotherapy [149]. In addition, combinations of other ICB, such as anti-TIM-3, or activating anti-CD137, confer long-term protection on ID8 tumor-bearing mice [150]. In most studies, mAb are administrated at the early stage of the disease (15 days in most ID8 models), and their efficacy in the late-stage disease, when ascites form, remains to be evaluated. ICB, the including PD-1/PD-L1 blockade, can produce synergistic antitumor effects, and significantly improve ID8 tumor-bearing mouse survival, when combined with chemotherapy, such as paclitaxel, which is able to upregulate PD-L1 expression in tumor cells [151], or trabectedin [152]. Carboplatin, however, despite its ability to increase CD4^+^ and CD8^+^ T-cell, and decrease Treg and MDSC, infiltration into tumors, does not appear to improve the effect of PD-1/PD-L1 blockades [153]. Other agents, aiming to modify the tumor immune context, were used in order to improve ICB efficacy. For instance, the stimulator of interferon genes (STING) agonists increases antigen presentation, CD8^+^ T-cell infiltration and IFN response in ID8 tumors, when combined with anti-PD-1, hence drastically improving mouse survival [154]. Decitabine, a DNA methyl transferase inhibitor, improves the antitumor effect of anti-CTLA-4 treatment, by extending survival in friend leukemia virus B (FVB) mice IP-injected with the BR5FVB1-Akt ovarian cancer cell line. Indeed, the combination triggers memory T-cell infiltration, and the production of cytokines related to effector CD8^+^ T-cells and NK cells in the peritoneal fluid [155]. PARP inhibitors (PARPi), such as veliparib [156] or niraparib [157], can increase the efficacy of anti-CTLA-4 or anti-PD-1 therapies, respectively, through the type II IFN, as well as the memory T-cell–mediated, antitumor effects in both cases.

Adoptive T-cell therapy (ACT): ACT involves the ex vivo selection of naturally-arising autologous antigen-specific T-cells, or the in vitro generation of specific T-cells, by transducing bulk autologous T-cells with viruses encoding modified TCR, specific for tumor Ag-derived epitopes. These cells are then expanded and re-infused in patients in order to achieve tumor targeting. In the ID8 model, the transfer of naïve T-cells, primed in vitro against ID8 Ag, combined with the depletion of tumor-associated immunosuppressive DC, results in durable rejection of EOC [158]. In this study, the therapeutic activity required the expression of perforin and CCL5 by adoptively-transferred T-cells [158]. T-cells can also be genetically modified to express a chimeric antigen receptor (CAR), encoding the tumor Ag-binding domain of an immunoglobulin linked to T-cell costimulatory molecules [159]. Preclinical studies, using humanized xenografted mouse models, assessed the activity of CAR T-cells against several human EOC Ags. For instance, Brentjens’s group used human EOC-bearing SCID-Beige mice to demonstrate the therapeutic efficacy of adoptively transferred CAR T-cells, targeting the extracellular conserved domain of MUC16 (MUC16^ecto^) [160]. More recently, the preclinical antitumor efficacy of MUC16^ecto^-targeting CAR T-cells was improved by genetically modifying them to produce IL-12 [161]. Other proteins highly expressed in EOC were recently targeted by CAR T-cells, including mesothelin [162], folate receptor (FR) [163], 5T4 oncofetal Ag [164] and B7-H3 [165], thus effectively controlling tumor growth in ID8 or human EOC-grafted mice. Other preclinical studies demonstrated the antitumor efficacy of CAR T-cells directed against adhesion molecules-expressing cells, including EpCAM [166], αvβ6 integrin [167] and L1-CAR [168], in human EOC-xenografted mice, which reported tumor regression and significantly prolonged survival.

Immunosuppressive microenvironment targeting: Targeting ovarian tumor-infiltrating immunosuppressive cell subsets is a potential strategy for opposing EOC tumor progression. For instance, immunosuppressive TAM limit the efficacy of antitumorigenic thymoquinone therapy, and their combination with the macrophage-depleting drug liposomal clodronate restores treatment efficacy in the ID8 model [169]. Targeting the CSF-1 receptor (CSF-1R) pathway, which is central to macrophage differentiation and survival, reduces TAM infiltration in ascites [170], and overcomes treatment resistance when combined with anti-VEGF [171]. Monocyte recruitment can be prevented through the targeting of chemokines, such as CCL2. Trabectedin, a DNA-damaging alkaloid, was found to inhibit CCL2 and IL-6 production, exerting selective toxicity for TAM in an EOC mouse model [172]. Another strategy to deplete TAM consists of exploiting their phenotype, which potentially includes the elevated expression levels of folate receptor-2 (FR2) in human and murine EOC. Indeed, FOLR2^+^ TAM can be effectively depleted, in preclinical ovarian tumors and ascites, using G5-methotrexate nanoparticles [173]. Vascular leukocytes and Tie2^+^ monocytes express high levels of CD52, and can be targeted using the anti-CD52 mAb Alemtuzumab. This therapy exhibits potent anti-myeloid and anti-angiogenic properties, thus restricting EOC growth in the ID8 model [174]. TAM can also be re-polarized, from regulatory M2 to antitumor M1, through NF-κB targeting, thus favoring Th1 cytokine productions and inhibiting tumor growth in mice [175]. Zhang et al. described nanoparticles that can deliver an in vitro-transcribed mRNA, encoding M1-polarizing transcription factors, IFN regulatory factor 5, and activating kinase IKKβ. In an ID8 model, it was observed that infusion of the nanoparticles reprograms TAM to an antitumor phenotype, which induces antitumor immunity and promotes tumor regression [176]. Natural plant-derived products, such as neferine, also inhibit M2 polarization in the EOC xenograft mouse model [177]. Finally, epigenetic modulators, such as bromodomain inhibitor JQ1, were demonstrated to prevent myeloid cell-related immunosuppression, by significantly reducing PD-L1 expression in TAM and DC, thus inducing increased T-cell cytotoxic activity and reducing ovarian tumor growth [178]. Zahnow’s group observed that combining DNA methlytransferase inhibitors with histone deacetylase inhibitors, or with an ornithine decarboxylase inhibitor, can reduce M2 infiltration in tumors, while increasing tumor-killing M1, T- and NK cell activation, thus delaying EOC progression in an ID8 VEGF-expressing mouse model [179,180]. Other immunosuppressive cells, such as Treg, that significantly infiltrate the EOC microenvironment, have been targeted in preclinical models. For instance, CCR4^+^ Treg recruitment was targeted by Chang et al., who administrated anti-CCR4, in a NOD scid gamma (NSG) humanized mouse model bearing human CCL22-secreting EOC, and engendered the potent restoration of antitumor immunity [181]. Righi et al. proposed the modulation of the CXCL12–CXCR4 axis, involved in Treg and MDSC recruitment, through the use of a selective CXCR4 antagonist. They were able to increase tumor apoptosis and necrosis, and reduce intratumoral Treg, in a syngeneic EOC mouse model [182]. The same group demonstrated that combining the CXCL12–CXCR4 axis blockade with anti-PD-1 further improves the antitumor effect of the CXCR4 antagonist [183].

Cytokines-based therapies: Potent immunomodulatory cytokines, including IL-12, IL-2, IFN-α and IFN-γ, are known to enhance antitumor immune responses, and some can exert direct cytotoxicity in EOC [184]. Initial studies in EOC mouse models highlighted an in vivo antitumor effect following the direct repeated injection of recombinant IL-12 [185], and underlined that IL-12 potently induces INF-γ, which stimulates tumor infiltration by lymphoid cells [186]. Later, liposomal delivery of IL-12 [187], and gene-based therapy delivery of IL-12 [188], proved efficient in EOC preclinical models. Other strategies of IL-12 delivery were validated as being able to induce strong antitumor effects, such as through IL-12-secreting transformed fibroblasts in the ID8 model [189], or through an armed-oncolytic herpes simplex virus in a GEMM developing spontaneous EOC [190]. The in vivo transduction of IL-12- and CCL27-encoding genes into preexisting murine EOC induced tumor regression and the development of long-term specific immunity [191]. Bankert et al. developed a humanized PDX-NSG mouse model, and evaluated the efficacy of human IL-12-loaded liposomes as a potential immunotherapy for EOC. Following treatment, they observed activation of T- and NK cells among TIL, leading to increased IFN-γ production [192]. IFN-based therapies were also initially tested via direct intraperitoneal injection in EOC preclinical models [193]. IFN-α is able to boost the antitumor effect of paclitaxel [194]. In addition, IFN-γ- and IFN-α2a-based therapy synergizes with monocyte adoptive transfer, mediating a profound antitumor effect in EOC-xenografted immunodeficient mice [195]. Nonetheless, caution may be necessary when using IFN-γ in particular, as it is known to upregulate suppressive PD-L1 in EOC cells [146], thus potentially promoting OC progression [196].

Vaccines: As inducers of specific adaptive immune responses, vaccines are promising for antitumor immunotherapy [197]. Therapeutic anticancer vaccines aim to boost or prime adaptive immune responses by delivering tumor Ag, and can be classified in different categories, such as cell-based, peptide/protein, epigenetic and genetic vaccines [197]. Despite its low mutational burden, the ID8-EOC mouse model was used to design neoantigen-targeting peptide vaccines. In this model, however, although vaccination with 17 synthetic peptides, covering the potential neoantigens, induced CD4^+^ or CD8^+^ T-cell responses to 7 mutated peptides, no survival benefit was observed [198]. This could be due to the lack of appropriate processing and presentation of these epitopes in tumor cells, or to the low avidity of the vaccine-induced T-cells, that were, as shown by the authors, not able to recognize tumor cells. Cell-based vaccines can use DC pulsed with whole tumor lysates, thus covering the complete repertoire of tumor Ag. Coukos’ group demonstrated the preclinical efficacy of DC, pulsed with hypochlorous acid (HOCl)-oxidized ID8-OVA whole tumor lysate, in eliciting an IFN-γ-dependent specific antitumor response, and in controlling tumor progression [141]. GEMM mice are useful in assessing vaccine efficacy using human tumor Ag. For instance, mice expressing human MUC1, and exhibiting *Pten* deletion and an activating *Kras* mutation, spontaneously develop ovarian tumors highly infiltrated by FOXP3^+^ Treg and dysfunctional DC [199]. In this model, vaccination with type 1-polarized DC loaded with MUC1 peptides reduces Treg infiltration and extends survival [199]. In another study, Chang et al. assessed the efficacy of a cell-based vaccine targeting mesothelin, an EOC Ag, in combination with an IL-12-encoding virus, and demonstrated enhanced specific CD4^+^ and CD8^+^ T-cells, and prolonged survival [200]. Other types of vaccines, such as DNA or RNA vaccines, used to induce the in vivo expression of selected Ag in cells, such as DC, infiltrating the vaccination site, proved efficient in preclinical cancer models (mainly in tumor types other than ovarian cancer) [197,201,202]. However, vaccines are currently being tested in clinical trials for the treatment of EOC patients [203].

## 4. Clinical Status of Immunotherapy Efficacy in Ovarian Cancer

### 4.1. Immune Checkpoint Blockade Monotherapy

The results from several ICB phase I/II clinical trials in ovarian cancer showed overall response rates (ORR) of 10–15% for recurrent ovarian cancer, including heavily treated and platinum-resistant patients, in most studies (Table 1). Complete and durable responses have been found in a minority of patients, partial responses in up to 10%, and stable disease in approximately 30% of cases. Median progression free survival (PFS) varies between 2 and 3.5 months, and OS between 17 and 20 months [204,205,206,207,208,209]. Grades 3 and 4 toxicity are observed in 7–40% of patients, and immune-related events in 16.8–22.6%. It is unclear whether PD-L1 expression is associated with improved response rates in ovarian cancer. In one study, assessment of PD-L1 expression using a combined positive score (CPS), i.e., the determination of PD-L1 expression in tumor and immune cells, showed that such expression was significantly associated with response rate, with ORR of 4.1% for CPS < 1.5, and ORR of 10% for CPS ≥ 10 [204]. The ORR and survival outcome are significantly increased in patients with a longer platinum-free interval, treated with less than three lines of chemotherapy [204]. Improved patient selection and a combination with other approaches, in particular combinations with chemotherapy, PARPi and angiogenic inhibitors, aim to optimize ICB activity. 

Of interest, a mAb specific to carcinoembryonic Ag (CEA), that leads to tumor opsonization and elimination by antibody dependent cell-mediated cytotoxicity (ADCC) or complement-mediated cytotoxicity (CDC) [210], could actually have an innate ICB activity through the blockade of CEA’s interaction with carcinoembryonic antigen-related cell adhesion molecule 1 (CEACAM1), the latter of which enacts an inhibitory activity in NK cells [211]. NEO-201 enhances NK cell function, including cytokine production and ADCC in vitro. NEO-201 has been shown to decrease tumor growth in human pancreatic cancer xenograft models. A phase I dose escalation trial is currently underway, to assess safety in patients with solid tumors, including mucinous ovarian carcinoma (NCT03476681).

### 4.2. Immune Checkpoint Blockade Combination Strategies

PARP inhibitors and ICB: Maintenance treatment with PARPi has significantly improved the prognostic outcome of patients with platinum-sensitive ovarian cancer [212,213,214,215,216]. Homologous recombination deficiencies (HRD), including *BRCA1/BRCA2* and other DNA damage repair (DDR) gene mutations, occur in approximately half of HGSC patients, and motivate the use of non-conservative DNA repair mechanisms in altered cells, thus increasing the mutational load [217]. PARPi lead to synthetic lethality, whereby cell death results from the functional loss of two DDR proteins, one due to the germline or somatic tumor mutation responsible for HRD, and one to PARP inhibition [218]. In addition to the mutational burden, HRD is correlated with high TIL infiltration and PD-1 expression in EOC [219]. In addition, PARPi induce PD-L1 upregulation through increased IFN-γ expression [220]. Based on this rationale, several ongoing phase III trials are assessing the combination of ICB and PARPi in the frontline and recurrent settings (Table 2).

In platinum-sensitive recurrent disease, the phase II basket trial, MEDIOLA, evaluated the safety and efficacy of using Durvalumab (anti-PD-L1) and Olaparib (PARPi) in patients with germline *BRCA* mutations. The preliminary results show an ORR of 63%, including 19% complete responses (CR) and 44% partial responses (PR), and a low rate of adverse events (AEs) [221]. A phase I escalation study of Durvalumab and Olaparib, or Cediranib (anti-VEGFR), in recurrent EOC also showed improved disease control rates, compared to monotherapy strategies. Authors reported 17% and 50% PR rates, in the Olaparib and Cediranib arms, respectively [222]. In platinum-resistant disease, the phase I/II trial, TOPACIO/KEYNOTE-162, assessed Niraparib (PARPi) with Pembrolizumab (anti-PD-1) in 62 patients with recurrent EOC, irrespective of *BRCA* status. The ORR was 18%, with 5% CR, 13% PR, and 28% stable disease (SD). Median PFS was 3.4 months, and OS was not reached. High-grade immune-related AEs occurred in 6% of patients, anemia being the most common AE [223]. Results from both trails showed increased response rates to ICB using combination strategies with PARPi, even in *BRCA* non-mutated patients.

ICB and VEGF inhibitors: Antiangiogenic agents can promote normal vessel architecture, reduce hypoxia and improve antitumor immunity, by increasing CD8^+^ T-cell tumor infiltration, enhancing antigen presentation, or inducing TAM polarization to M1-subtype [224]. A dose-escalation phase I trial of Olaparib, Durvalumab and an anti-VEGF1-3, in the platinum-resistant recurrent setting, showed 44% PR [225]. As stated above, a dose-escalation phase I trial of Durvalumab showed the safety profile and activity for a combination with Olaparib or Cediranib [222]. Several ongoing phase III trials are assessing the efficacy of combination strategies using ICB and antiangiogenic agents in the frontline and recurrent settings (Table 2).

### 4.3. Vaccine Strategies

Targeting shared antigens: Aberrant overexpression of the folate binding protein or folate receptor alpha (FRα) is observed in more than 80% of ovarian cancers. FRα contributes to carcinogenesis through cell growth regulation and signaling functions, and has also been associated with chemotherapy resistance and poor prognosis [226]. Phase I/II FRα vaccine trials have demonstrated the low toxicity profile, and increased T-cell immunity, in small cohorts of recurrent ovarian cancer patients [227,228]. The best ORR were obtained after frontline treatment or in patients treated with less than 3 lines of chemotherapy [229,230]. Based on these results, a phase III trial has been conducted to assess PFS in platinum-resistant ovarian cancer patients with FRα expression, randomized to Mirvetuximab soravtansine (IMGN853) or standard chemotherapy (FORWARD I trial, NCT02631876).

In addition, several phase I trials have assessed vaccination strategies targeting P53, mutated in over 95% of HGSC, in platinum-resistant patients, and yielded rather unsatisfactory results [231,232,233]. A single study, nonetheless, using a modified recombinant vaccinia ankara vaccine, delivering wild type human p53 (p53MVA), in combination with gemcitabine chemotherapy found a significant association between P53-specific vaccine-induced CD4^+^ and CD8^+^ T-cell responses and PFS [233]. 

Cancer-testis Ag (CTA): Vaccination strategies targeting the CTA NY-ESO-1 have been developed in the clinic, using long peptides combined with adjuvants, recombinant viruses, or epigenetic modifiers. Published I/II trials have demonstrated the immunogenicity of long peptide/adjuvant and viral vector vaccines, which induced humoral and T-cell responses. However, most clinical trials included small numbers of patients, and were not initially designed to evaluate clinical efficacy [69,234,235]. A review, including a large cohort of cases, demonstrated the increased clinical responses of, and significant OS advantages for, patients with NY-ESO-1^+^ tumors treated with immunotherapy strategies targeting the Ag [236] (Table 3).

Targeting neoantigens: Recent early-phase clinical studies, performed in various cancer types, have demonstrated the possibility of enhancing neoantigen-specific T-cell responses with personalized vaccines [237,238]. However, only a small proportion of non-synonymous mutations will lead to antigenic peptides and tumor recognition by T-cells specific for these peptides [237]. Approaches to target neoantigens include vaccination with long peptides encompassing the mutation-encoded amino acid combined with adjuvants, and adoptive T-cell transfer. 

Although neoantigen vaccines have been preferentially developed in tumor types with a high mutational burden [237], neoantigen-specific T-cells are found in patients with low mutation load tumors, including EOC [66], and personalized neoantigen-based vaccines have been used in such tumor types [239]. Recently, personalized vaccination, using oxidized autologous whole-tumor lysates, was administered alone, in combination with bevacizumab, or with cyclophosphamide and bevacizumab, and was tested in a cohort of 25 patients with recurrent EOC until progression. The vaccine induced specific CD8^+^ and CD4^+^ polyfunctional T-cell responses, which were correlated to PFS. The vaccine amplified preexisting neo-epitope T-cell responses, and induced new responses to peptides harboring amino acids encoded by non-synonymous somatic mutations [240].

### 4.4. Adoptive T-Cell Transfer 

ACT trials have shown the objective and durable responses of several patients affected with various malignancies, in particular melanoma [241]. The efficacy of ACT has been explored in early-phase clinical trials for 20 years. Initial studies proposed a combination of IL-2-expanded TIL infusion or intraperitoneal administration with chemotherapy, resulting in variable response rates [242,243,244,245]. Later, studies in the clinic used targets, through TCR transgenic or CAR T-cell transfer approaches, in EOC, including CTA, FRα, MUC16, HER2, WT1 and p53 (Table 4). Multiple preclinical studies and some early-phase clinical trials have demonstrated the feasibility of this approach, and T-cell reactivity to targeted tumor antigens, with short-lived clinical responses. Possible explanations for this include the exhaustion of infused T-cells, antigen loss, MHC downregulation or loss, abnormal antigen presentation mechanisms, and immunosuppressive cytokines and cellular microenvironment [246]. The other limitations of ACT strategies include the ability to produce TCR transgenic or CAR T-cells, the lack of optimal targets, and high-grade toxicities. Cytokine release syndrome (CRS) can be associated with ACT secondary to T-cell activation and cytokine release, producing fever and major toxicity. In particular, one heavily treated patient with recurrent EOC received autologous mesothelin-directed CAR T-cells. The patient experienced a compartmental CRS in the pleural cavity, requiring treatment with an IL-6 receptor antagonist [247]. The first CAR T-cell experiment in EOC used autologous T-cells transduced to express an anti-FRα CAR. This phase I trial showed detectable gene-modified T-cells that nearly disappeared one month after infusion, and no tumor rejection was observed [248]. Due to the unique pattern of growth in the peritoneal cavity, and in order to reduce systemic toxicity, intraperitoneal perfusion has emerged as an alternative to systemic administration in ovarian cancer. The preliminary results of a dose escalation phase I trial demonstrate the feasibility of autologous IL-12-secreting, MUC-16-specific CAR T-cell administration through the intraperitoneal route [249].

### 4.5. Agents Targeting the Epigenome

Epigenome instability, including anomalies in DNA methylation (DNMT) and histone ubiquitination patterns, are related to ovarian cancer progression and platinum resistance [250]. The epigenetic silencing of genes involved in T helper responses, antigen presentation and processing have been reported. DNMT inhibitors have been shown to upregulate type I interferon signaling and apoptosis, through the viral response pathway and the endogenous retroviral gene transcripts in ovarian cancer [251]. DNMT and histone deacetylase inhibitors can reverse the immunosuppressive microenvironment by inducing MHC class I expression and CD8 T-cell responses [252]. DNA methylation of effector T-cell genes may be acquired in TILs, and can promote T-cell exhaustion by decreasing T-cell effector function, activation and proliferation. These exhaustion-associated DNA methylation alterations persist during ICB treatment, and are related to resistance [253]. DNMT inhibitors used in combination with ICB have been proven to reverse T-cell exhaustion-associated epigenetic patterns [253]. Agents targeting the epigenome have also been shown to induce platinum re-sensitization in resistant patients, via upregulation of tumor suppressor genes [254,255]. Early-phase trials using epigenetic approached are presented in Table 5.

## 5. Conclusions

Since the introduction of paclitaxel inro first-line treatment, no dramatic advances have been attained in EOC patient progression-free survival, eliciting high expectations in new therapeutic strategies, such as immunotherapy. Recent and increasing evidence demonstrates that EOC tumors are infiltrated by cytotoxic T lymphocytes, key actors of the antitumor immune response and predictors of patient outcome. Nonetheless, so far, the most promising immunotherapies, targeting immune checkpoints expressed by exhausted T-cells, have failed to prove effective in EOC. Their limitations are possibly due to a myriad of mechanisms, likely related to the microenvironment complexity, and including strong tumor immune evasion mechanisms. While EOC tumor microenvironment (TME) was examined in both preclinical and human contexts, more work is required to clarify EOC immune complexity, in order to guide immunotherapeutic options. 

Preclinical studies in EOC mouse models have helped us to understand the interactions between multiple cell subsets, including tumor cells and immune cells. In addition to tumor intrinsic qualities, such as immunogenicity, mutational burden, stage, and patient overall performance status and age, all components of the TME and their crosstalk need to be taken into account for the development of immunotherapy. Besides, a recent innovative concept involves the tumor’s anatomical location, including primary tumors, ascites and metastases, in determining the TME constitution, and thus in the global picture of treatment design. This concept appears particularly relevant in the context of EOC, considering the characteristic multi-location feature of the primary disease. 

Preclinical evidence has been paving the way to numerous immunotherapy trials in EOC patients, with many of them currently ongoing. To improve the clinical benefits, future trials should optimize patient selection, and future challenges will include evaluating synergistic combinations, that concomitantly increase antitumor response and dampen immunosuppressive pathways through the targeting of crucial TME components. 

## Figures and Tables

**Table 1:** Published results from selected ICB clinical trials for recurrent EOC.

Study	Phase	Drug	Population	N	ORR (%)	Stable Disease	PFS Months	OS Months	Grade 3/4 Toxicity (%)
**Anti-PD-1**									
Matulonis et al. [204]	II	Pembrolizumab	Refractory/recurrent OC Cohort A	376	8	29.3	2.1		19.7
			<3 lines, PFI 3–12 months Cohort B	285	7.4	10.5	2.1	NR	
			4–6 lines, PFI > 3 months	100	9.9	27.5	2.1	17.6 months	
Hamanishi et al. [206]	II	Nivolumab	Recurrent/persistent OC 1 to 3 lines PFI < 12 months	20	15	30	3.5	20	40
Zamarin et al. [207]	II	Nivolumab	Recurrent/persistent OC 1 to 3 lines	100	12	29	2	21.8	33
		Nivolumab and Ipilimumab	PFI < 12 months		31.4	39	3.9	28.1	49
**Anti-PD-L1**									
Disis et al. [205]	Ib	Avelumab	Recurrent OC >3 lines (64.8%)	125	9.6	42.4	10.2% at 12 months	11.2	7.2
Brahmer et al. [208]	I	BMS-936559	>1line	17	6	18	22% at 24 weeks		9
**Anti-CTAL4**									
Hodi et al. [209]	I	Ipilimumab	>1 line and GVAX vaccination (GM-CSF)	9	10	33			22

N: number of patients; ORR: overall response rate; PFS: progression-free survival; OS: overall survival; OC: ovarian cancer.

**Table 2:** Ongoing Phase III ICB, PARPi and/or Anti-VEGF trials.

Study	N	Arms	Primary Endpoint	Recruitment Status
**Frontline setting**				
GINECO FIRST-ENGOT Ov44 (NCT03740165)	912	Arm 1: CT + Bev + Placebo followed by Placebo Arm 2: CT + Placebo Followed by Placebo + Niraparib Arm 3: CT + Dostarlimab followed by Dostarlimab+Niraparib	PFS	Recruiting
AGO DUO-ENGOT (NCT03737643)	1056	Arm 1: CT + Bev + Placebo followed by Bev + Placebo Arm2: CT + Bev + Durvalumab followed by Bev + Durvalumab+Placebo Arm3: CT + Bev + Durvalumab followed by Bev + Durvalumab + Olaparib	PFS in non tBRCAmut	Recruiting
KELYNK-001 EBOGT-Ov43 (NCT03740165)	1086	Arm 1: CT + Placebo followed by Placebo +/- Bev Arm 2: CT + Pembrolizumab followed by Placebo +/- Bev Arm 3: CT+Pembrolizumab followed by Pembrolizumab + Olaparib +/- Bev	PFS and OS	Recruiting
ATHENA (NCT03522246)	1012	Arm 1: CT followed by Placebo Arm 2: CT followed by Rucaparib + Placebo Arm 3: CT followed by Nivolumab + Placebo Arm 4: CT followed by Rucaparib + Nivolumab	PFS	Recruiting
IMagyn 50 GOG3015-ENGOT 0v39 (NCT 03038100)	1300	Arm 1: CT + Bev + Placebo Arm 2: CT+ Bev+ Atezolizumab	PFS, OS PSF and OS assessed by PD-L1	Active Not recruiting
**Recurrent platinum-sensitive disease**				
ATALANTE/ENGOT Ov29 (NCT02891824)	405	Arm1: Placebo + Bev + platinum-based chemotherapy. Arm 2: Atezolizumab + Bev + platinum-based chemotherapy	PFS	Active, not recruiting
ANITA/GEICO 69/ENGOT Ov41 (NCT03598270)	414	Arm 1: Carboplatin + Niraparib Arm 2: Carboplatin + Niraparib + Atezolizumab	PFS	Recruiting
**Recurrent platinum resistant**				
EORTC -1508 (NCT02659384)	160	Arm 1: Bev + Atezolizumab Arm 2: Bev + Atezolizumab + aspririn	PFS at 6 months	Recruiting

CT: Carboplatin-taxol; Bev: Bevacizumab; PFS: progression-free survival; OS: overall survival.

**Table 3:** Selected ongoing vaccination trials.

Study (NCT)	Phase	Type of Vaccine	N	Population	Primary Objective	Recruitment Status
NCT01536054	I	ALVAC(2)-NY-ESO-1 (M)/TRICOM vaccine with sirolimus	42	Stage II–IV ovarian, FT or primary peritoneal cancer	Safety	Completed
NCT00616941	I	NY-ESO-1 OLP4 + Montanide + Poly-ICLC	28	Stage II–IV and recurrent disease ovarian, FT or primary peritoneal cancer	Safety	Completed
NCT00112957	I/II	Recombinant vaccinia-NY-ESO-1 (rV-NY-ESO-1) and recombinant fowlpox-NY-ESO-1 (rF-NY-ESO-1)	23	Ovarian, FT or primary peritoneal cancer with NY-ESO-1 or LAGE-1 expression	12 months DFS	Completed
NCT02166905	I	IDO1 inhibitor INCB024360 in combination with DEC-205/NY-ESO-1 fusion protein CDX-1401 and poly ICLC	62	Ovarian, FT or primary peritoneal cancer in remission after CT for primary or recurrent disease	Safety Toxicity PFS	Recruiting
NCT00948961	I/II	CDX-1401 in combination with Resiquimod and/or Poly-ICLC	70	Ovarian cancer and other solid tumors with NY-ESO-1 expression	Safety	Completed
NCT01673217	I	Decitabine, NYESO-I protein mixed with montanide and (GM-CSF)	18	Recurrent ovarian, FT or primary peritoneal cancer	Safety	Completed
NCT02432378	I/II	Cisplatin + CKM + Celecoxib + DC intranodal vaccine	25	Recurrent platinum-sensitive ovarian cancer	Safety, CD8 in the peritoneal cavity	Recruiting
NCT03029403	II	DPX-Survivac Vaccine+pembrolizumab + cyclophosphamide	42	Ovarian, FT or primary peritoneal cancer after first line	ORR	Recruiting
NCT02759588	I/II	GL-ONC1 +/− chemotherapy +/− Bevacizumab	64	Resistant/refractory	Safety, PFS, Ca125, ORR	Recruiting

FT: fallopian tube; DFS: Disease-free survival; IDO1: Indoleamine-pyrrole 2,3-dioxygenase; polyICLC: Polyinosinic-Polycytidylic acid with Polylysine and Carboxymethylcellulose; CT: chemotherapy; ORR: overall response rate; Ca125: carbohydrate antigen 125.

**Table 4:** Selected ongoing adoptive T-cell trials.

Target	Study (NCT)	Phase	N	Population	Protocol	Primary objective	Recruitment Status
**MUC-16**	NCT02498912	I	30	Recurrent MUC16 + solid tumors	Cyclophosphamide followed by Iv ip infusion of MUC16 specific T-cells secreting IL-12	Safety	Recruiting
**HER2**	NCT00194714	I/II	26	HLA2 Stage IV HER2 + breast/OC	HER2 peptide vaccination	Safety	Active, not recruiting
	NCT00228358	I	8	Metastatic HER2 tumors previously vaccinated	Cyclophosphamide or denileukin diftitox followed by Expanded HER2-specific T-cells	Feasibility Safety	Completed
**WT1**	NCT00562640	I	21	Recurrent WT + OC, FT, PPC	WT-1 specific T-cells	Safety and tolerability	Active, not recruiting
**Mesothelin**	NCT02580747	II	20	Refractory recurrent mesothelin+ OC	Anti-meso-CAR T-cells	Safety, feasibility. Duration meso-	Unknown
	NCT03054298	I	30	Recurrent mesothelin+ OC	huCART-meso cells	CAR T-cells Safety, feasibility	Active, not recruiting Terminated
	NCT01583686	I	136	Metastatic mesothelin + cancer	Lymphodepletion followed by Anti-meso-CAR T-cells	Safety, ORR	Completed
	NCT02159716	I	24	Metastatic mesothelin + cancer	Anti-meso-CAR T-cells +/-cyclophosphamide	Safety and feasibility	
**CD133**	NCT02541370	I	20	CD-33 + Refractory Cancers	Anti-CD133-CAR T-cells	Safety	Completed
**MAGE-A4**	NCT03132922	I	42	HLA2+ with MAGE-A4 cancer Persistent disease	Anti-MAGE-A4^c^¹º³² T-cells	Safety, duration of Anti-MAGE4 T-cells	Recruiting
**NY-ESO-1**	NCT03159585	I	20	HLA-A 0201+ patients with solid tumors NYESO-1+	NY-ESO-1- T-cells (TAEST16001)	Safety	Completed
	NCT01567891	I/II	9	HLA A 0201, HLA-A 0205, and/or HLA-A 0206 recurrent OC < 2lines	NYESO-1c259 T-cells	Safety	Completed
	NCT03017131	I	12	Recurrent/refractory OC	Autologous NY-ESO-1 T-cell + decitabine + IL-2	Safety and tolerability	Recruiting
	NCT00101257	I	18	HLA DPB 0401, DPB1 0201, DRB1 07 with Stage III/IV	Autologous CD4-positive antigen-specific T-cells	Safety, toxicity, duration in vivo infused T-cells	Recruiting
	NCT02166905	I/IIb	64	NYESO-1+OC NY-ESO-1 or LAGE-1 + primary or recurrent OC	EC-205/NY-ESO-1 Fusion Protein CDX-1401, Poly ICLC, and IDO1 Inhibitor INCB024360	Safety, PFS	Recruiting
**Neoantigens**	NCT03412877	I/II	210	Metastatic/refractory solid cancer with measurable disease	Autologous T-cells engineered to express TCR Anti-Neoantigens	ORR	Recruiting

MUC: mucin; HER2: human epidermal growth factor receptor 2; OC: ovarian cancer; FT; fallopian tube; PPC: primary peritoneal cancer; WT1: Wilms Tumor; MAGE-A4: melanoma antigen gene family; NY-ESO-1: New York esophageal carcinoma.

**Table 5:** Clinical trials with agents targeting epigenome.

Target	Study (NCT)	Phase	N	Population	Protocol	Primary Objective	Results
Decitabine	NCT00887796	I	12	Recurrent OC	Decitabine+ NYESO-I protein with montanide + GM-CSF+ liposomal doxorubicin	Safety	Well-tolerated DNA Hypomethylation/ blood, circulating DNAs NY-ESO-1 Ab and T-cell responses SD 50%, PR 10%
Entinostat	NCT02915523	I/II	140	Recurrent OC >2 lines CT	Avelumab With or Without Entinostat	Dose Efficacy (PFS)	NR
Guadecitabine	NCT02901899	II	38	Recurrent OC > 1 ≤ 3 lines	Guadecitabine + Pembrolizumab	ORR	NR
Guadecitabine	NCT03206047	I/IIb	75	Platinum-resistant recurrent OC	Atezolizumab, Guadecitabine, and CDX-1401 Vaccine	Safety Efficacy (PFS)	NR
Guadecitabine SGI-110-02	NCT01696032	I/II	100	Platinum-resistant recurrent OC	SGI-110-02 + Carboplatin or CT	Efficacy (PFS)	6-month PFS rate increased in the Guadecitabine group (37% vs. 11%)

OC: ovarian cancer; CT: chemotherapy; GM-CSF: granulocyte-macrophage colony stimulating factor; SD: stable disease; PR: partial response; NR: not reported

## References

[1] Cannistra S.A. (2004). Cancer of the ovary. N. Engl. J. Med..

[2] Bast R.C., Hennessy B., Mills G.B. (2009). The biology of ovarian cancer: New opportunities for translation. Nat. Rev. Cancer.

[3] Kurman R.J., Shih I.M. (2010). The origin and pathogenesis of epithelial ovarian cancer: A proposed unifying theory. Am. J. Surg. Pathol..

[4] Kossaï M., Leary A., Scoazec J.Y., Genestie C. (2018). Ovarian Cancer: A Heterogeneous Disease. Pathobiol. J. Immunopathol. Mol. Cell. Biol..

[5] Zhang L., Conejo-Garcia J.R., Katsaros D., Gimotty P.A., Massobrio M., Regnani G., Makrigiannakis A., Gray H., Schlienger K., Liebman M.N. (2003). Intratumoral T-cells, recurrence, and survival in epithelial ovarian cancer. N. Engl. J. Med..

[6] Hwang W.T., Adams S.F., Tahirovic E., Hagemann I.S., Coukos G. (2012). Prognostic significance of tumor-infiltrating T-cells in ovarian cancer: A meta-analysis. Gynecol. Oncol..

[7] Jiménez-Sánchez A., Memon D., Pourpe S., Veeraraghavan H., Li Y., Vargas H.A., Gill M.B., Park K.J., Zivanovic O., Konner J. (2017). Heterogeneous Tumor-Immune Microenvironments among Differentially Growing Metastases in an Ovarian Cancer Patient. Cell.

[8] Kandalaft L.E., Motz G.T., Duraiswamy J., Coukos G. (2011). Tumor immune surveillance and ovarian cancer: Lessons on immune mediated tumor rejection or tolerance. Cancer Metastasis Rev..

[9] Topalian S.L., Hodi F.S., Brahmer J.R., Gettinger S.N., Smith D.C., McDermott D.F., Powderly J.D., Carvajal R.D., Sosman J.A., Atkins M.B. (2012). Safety, activity, and immune correlates of anti-PD-1 antibody in cancer. N. Engl. J. Med..

[10] Fridman W.H., Zitvogel L., Sautès-Fridman C., Kroemer G. (2017). The immune contexture in cancer prognosis and treatment. Nat. Rev. Clin. Oncol..

[11] Fridman W.H., Pagès F., Sautès-Fridman C., Galon J. (2012). The immune contexture in human tumours: Impact on clinical outcome. Nat. Rev. Cancer.

[12] Galon J., Costes A., Sanchez-Cabo F., Kirilovsky A., Mlecnik B., Lagorce-Pagès C., Tosolini M., Camus M., Berger A., Wind P. (2006). Type, density, and location of immune cells within human colorectal tumors predict clinical outcome. Science.

[13] Montfort A., Owen S., Piskorz A.M., Supernat A., Moore L., Al-Khalidi S., Böhm S., Pharoah P., McDermott J., Balkwill F.R. (2020). Combining measures of immune infiltration shows additive effect on survival prediction in high-grade serous ovarian carcinoma. Br. J. Cancer.

[14] Thorsson V., Gibbs D.L., Brown S.D., Wolf D., Bortone D.S., Ou Yang T.H., Porta-Pardo E., Gao G.F., Plaisier C.L., Eddy J.A. (2018). The Immune Landscape of Cancer. Immunity.

[15] Salmon H., Remark R., Gnjatic S., Merad M. (2019). Host tissue determinants of tumour immunity. Nat. Rev. Cancer.

[16] Devaud C., Westwood J.A., John L.B., Flynn J.K., Paquet-Fifield S., Duong C.P.M., Yong C.S.M., Pegram H.J., Stacker S.A., Achen M.G. (2014). Tissues in different anatomical sites can sculpt and vary the tumor microenvironment to affect responses to therapy. Mol. Ther. J. Am. Soc. Gene Ther..

[17] Devaud C., John L.B., Westwood J.A., Yong C.S., Beavis P.A., Schwendener R.A., Darcy P.K., Kershaw M.H. (2015). Cross-talk between tumors can affect responses to therapy. Oncoimmunology.

[18] Trimaglio G., Tilkin-Mariamé A.F., Feliu V., Lauzeral-Vizcaino F., Tosolini M., Valle C., Ayyoub M., Neyrolles O., Vergnolle N., Rombouts Y. (2020). Colon-specific immune microenvironment regulates cancer progression versus rejection. bioRxiv.

[19] Van den Eynde M., Mlecnik B., Bindea G., Fredriksen T., Church S.E., Lafontaine L., Haicheur N., Marliot F., Angelova M., Vasaturo A. (2018). The Link between the Multiverse of Immune Microenvironments in Metastases and the Survival of Colorectal Cancer Patients. Cancer Cell.

[20] Vesely M.D., Kershaw M.H., Schreiber R.D., Smyth M.J. (2011). Natural innate and adaptive immunity to cancer. Annu. Rev. Immunol..

[21] Mittal D., Gubin M.M., Schreiber R.D., Smyth M.J. (2014). New insights into cancer immunoediting and its three component phases--elimination, equilibrium and escape. Curr. Opin. Immunol..

[22] Dadmarz R.D., Ordoubadi A., Mixon A., Thompson C.O., Barracchini K.C., Hijazi Y.M., Steller M.A., Rosenberg S.A., Schwartzentruber D.J. (1996). Tumor-infiltrating lymphocytes from human ovarian cancer patients recognize autologous tumor in an MHC class II-restricted fashion. Cancer J. Sci. Am..

[23] Naito Y., Saito K., Shiiba K., Ohuchi A., Saigenji K., Nagura H., Ohtani H. (1998). CD8+ T-cells infiltrated within cancer cell nests as a prognostic factor in human colorectal cancer. Cancer Res..

[24] Ma D., Gu M.J. (1991). Immune effect of tumor-infiltrating lymphocytes and its relation to the survival rate of patients with ovarian malignancies. J. Tongji Med. Univ..

[25] Leffers N., Gooden M.J.M., de Jong R.A., Hoogeboom B.N., ten Hoor K.A., Hollema H., Boezen H.M., van der Zee A.G.J., Daemen T., Nijman H.W. (2009). Prognostic significance of tumor-infiltrating T-lymphocytes in primary and metastatic lesions of advanced stage ovarian cancer. Cancer Immunol. Immunother..

[26] Nielsen J.S., Sahota R.A., Milne K., Kost S.E., Nesslinger N.J., Watson P.H., Nelson B.H. (2012). CD20+ tumor-infiltrating lymphocytes have an atypical CD27- memory phenotype and together with CD8+ T-cells promote favorable prognosis in ovarian cancer. Clin. Cancer Res. Off. J. Am. Assoc. Cancer Res..

[27] Hermans C., Anz D., Engel J., Kirchner T., Endres S., Mayr D. (2014). Analysis of FoxP3+ T-regulatory cells and CD8+ T-cells in ovarian carcinoma: Location and tumor infiltration patterns are key prognostic markers. PLoS ONE.

[28] Bronger H., Singer J., Windmüller C., Reuning U., Zech D., Delbridge C., Dorn J., Kiechle M., Schmalfeldt B., Schmitt M. (2016). CXCL9 and CXCL10 predict survival and are regulated by cyclooxygenase inhibition in advanced serous ovarian cancer. Br. J. Cancer.

[29] Webb J.R., Milne K., Watson P., Deleeuw R.J., Nelson B.H. (2014). Tumor-infiltrating lymphocytes expressing the tissue resident memory marker CD103 are associated with increased survival in high-grade serous ovarian cancer. Clin. Cancer Res. Off. J. Am. Assoc. Cancer Res..

[30] Komdeur F.L., Wouters M.C.A., Workel H.H., Tijans A.M., Terwindt A.L.J., Brunekreeft K.L., Plat A., Klip H.G., Eggink F.A., Leffers N. (2016). CD103+ intraepithelial T-cells in high-grade serous ovarian cancer are phenotypically diverse TCRαβ+ CD8αβ+ T-cells that can be targeted for cancer immunotherapy. Oncotarget.

[31] St Paul M., Saibil S.D., Lien S.C., Han S., Sayad A., Mulder D.T., Garcia-Batres C.R., Elford A.R., Israni-Winger K., Robert-Tissot C. (2020). IL6 Induces an IL22+ CD8+ T-cell Subset with Potent Antitumor Function. Cancer Immunol. Res..

[32] Durgeau A., Virk Y., Corgnac S., Mami-Chouaib F. (2018). Recent Advances in Targeting CD8 T-Cell Immunity for More Effective Cancer Immunotherapy. Front. Immunol..

[33] Van der Leun A.M., Thommen D.S., Schumacher T.N. (2020). CD8+ T-cell states in human cancer: Insights from single-cell analysis. Nat. Rev. Cancer.

[34] Balança C.C., Scarlata C.M., Michelas M., Devaud C., Sarradin V., Franchet C., Martinez Gomez C., Gomez-Roca C., Tosolini M., Heaugwane D. (2020). Dual Relief of T-Lymphocyte Proliferation and Effector Function Underlies Response to PD-1 Blockade in Epithelial Malignancies. Cancer Immunol. Res..

[35] Matsuzaki J., Gnjatic S., Mhawech-Fauceglia P., Beck A., Miller A., Tsuji T., Eppolito C., Qian F., Lele S., Shrikant P. (2010). Tumor-infiltrating NY-ESO-1-specific CD8+ T-cells are negatively regulated by LAG-3 and PD-1 in human ovarian cancer. Proc. Natl. Acad. Sci. USA.

[36] Whelan S., Ophir E., Kotturi M.F., Levy O., Ganguly S., Leung L., Vaknin I., Kumar S., Dassa L., Hansen K. (2019). PVRIG and PVRL2 Are Induced in Cancer and Inhibit CD8+ T-cell Function. Cancer Immunol. Res..

[37] Sawada M., Goto K., Morimoto-Okazawa A., Haruna M., Yamamoto K., Yamamoto Y., Nakagawa S., Hiramatsu K., Matsuzaki S., Kobayashi E. (2020). PD-1+ Tim3+ tumor-infiltrating CD8 T-cells sustain the potential for IFN-γ production, but lose cytotoxic activity in ovarian cancer. Int. Immunol..

[38] Valmori D., Ayyoub M. (2015). CD4+ T helper cell responses to NY-ESO-1 tumor antigen in ovarian cancer resist perversion into immunosuppressive Tregs. Oncoimmunology.

[39] Hamanishi J., Mandai M., Abiko K., Matsumura N., Baba T., Yoshioka Y., Kosaka K., Konishi I. (2011). The comprehensive assessment of local immune status of ovarian cancer by the clustering of multiple immune factors. Clin. Immunol..

[40] Tsiatas M.L., Gyftaki R., Liacos C., Politi E., Rodolakis A., Dimopoulos M.A., Bamias A. (2009). Study of T lymphocytes infiltrating peritoneal metastases in advanced ovarian cancer: Associations with vascular endothelial growth factor levels and prognosis in patients receiving platinum-based chemotherapy. Int. J. Gynecol. Cancer Off. J. Int. Gynecol. Cancer Soc..

[41] Le Page C., Marineau A., Bonza P.K., Rahimi K., Cyr L., Labouba I., Madore J., Delvoye N., Mes-Masson A.M., Provencher D.M. (2012). BTN3A2 expression in epithelial ovarian cancer is associated with higher tumor infiltrating T-cells and a better prognosis. PLoS ONE.

[42] DeLeeuw R.J., Kroeger D.R., Kost S.E., Chang P.P., Webb J.R., Nelson B.H. (2015). CD25 identifies a subset of CD4^+^FoxP3^−^ TIL that are exhausted yet prognostically favorable in human ovarian cancer. Cancer Immunol. Res..

[43] Redjimi N., Duperrier-Amouriaux K., Raimbaud I., Luescher I., Dojcinovic D., Classe J.M., Berton-Rigaud D., Frenel J.S., Bourbouloux E., Valmori D. (2011). NY-ESO-1-specific circulating CD4+ T-cells in ovarian cancer patients are prevalently T(H)1 type cells undetectable in the CD25+ FOXP3+ Treg compartment. PLoS ONE.

[44] Ayyoub M., Dojcinovic D., Pignon P., Raimbaud I., Schmidt J., Luescher I., Valmori D. (2010). Monitoring of NY-ESO-1 specific CD4+ T-cells using molecularly defined MHC class II/His-tag-peptide tetramers. Proc. Natl. Acad. Sci. USA.

[45] Ayyoub M., Pignon P., Classe J.M., Odunsi K., Valmori D. (2013). CD4+ T effectors specific for the tumor antigen NY-ESO-1 are highly enriched at ovarian cancer sites and coexist with, but are distinct from, tumor-associated Treg. Cancer Immunol. Res..

[46] Nesbeth Y.C., Martinez D.G., Toraya S., Scarlett U.K., Cubillos-Ruiz J.R., Rutkowski M.R., Conejo-Garcia J.R. (2010). CD4+ T-cells elicit host immune responses to MHC class II-negative ovarian cancer through CCL5 secretion and CD40-mediated licensing of dendritic cells. J. Immunol..

[47] Kryczek I., Banerjee M., Cheng P., Vatan L., Szeliga W., Wei S., Huang E., Finlayson E., Simeone D., Welling T.H. (2009). Phenotype, distribution, generation, and functional and clinical relevance of Th17 cells in the human tumor environments. Blood.

[48] Charles K.A., Kulbe H., Soper R., Escorcio-Correia M., Lawrence T., Schultheis A., Chakravarty P., Thompson R.G., Kollias G., Smyth J.F. (2009). The tumor-promoting actions of TNF-alpha involve TNFR1 and IL-17 in ovarian cancer in mice and humans. J. Clin. Investig..

[49] Winkler I., Pyszniak M., Pogoda K., Semczuk A., Gogacz M., Miotla P., Adamiak A., Darmochwal-Kolarz D., Rechberger T., Tabarkiewicz J. (2017). Assessment of Th17 lymphocytes and cytokine IL-17A in epithelial ovarian tumors. Oncol. Rep..

[50] Bilska M., Pawłowska A., Zakrzewska E., Chudzik A., Suszczyk D., Gogacz M., Wertel I. (2020). Th17 Cells and IL-17 As Novel Immune Targets in Ovarian Cancer Therapy. J. Oncol..

[51] Wing J.B., Tanaka A., Sakaguchi S. (2019). Human FOXP3+ Regulatory T-cell Heterogeneity and Function in Autoimmunity and Cancer. Immunity.

[52] Woo E.Y., Chu C.S., Goletz T.J., Schlienger K., Yeh H., Coukos G., Rubin S.C., Kaiser L.R., June C.H. (2001). Regulatory CD4(+)CD25(+) T-cells in tumors from patients with early-stage non-small cell lung cancer and late-stage ovarian cancer. Cancer Res..

[53] Sehouli J., Loddenkemper C., Cornu T., Schwachula T., Hoffmüller U., Grützkau A., Lohneis P., Dickhaus T., Gröne J., Kruschewski M. (2011). Epigenetic quantification of tumor-infiltrating T-lymphocytes. Epigenetics.

[54] Kryczek I., Wei S., Zhu G., Myers L., Mottram P., Cheng P., Chen L., Coukos G., Zou W. (2007). Relationship between B7-H4, regulatory T-cells, and patient outcome in human ovarian carcinoma. Cancer Res..

[55] Wolf D., Wolf A.M., Rumpold H., Fiegl H., Zeimet A.G., Muller-Holzner E., Deibl M., Gastl G., Gunsilius E., Marth C. (2005). The expression of the regulatory T-cell-specific forkhead box transcription factor FoxP3 is associated with poor prognosis in ovarian cancer. Clin. Cancer Res. Off. J. Am. Assoc. Cancer Res..

[56] Curiel T.J., Coukos G., Zou L., Alvarez X., Cheng P., Mottram P., Evdemon-Hogan M., Conejo-Garcia J.R., Zhang L., Burow M. (2004). Specific recruitment of regulatory T-cells in ovarian carcinoma fosters immune privilege and predicts reduced survival. Nat. Med..

[57] Fialová A., Partlová S., Sojka L., Hromádková H., Brtnický T., Fučíková J., Kocián P., Rob L., Bartůňková J., Spíšek R. (2013). Dynamics of T-cell infiltration during the course of ovarian cancer: The gradual shift from a Th17 effector cell response to a predominant infiltration by regulatory T-cells. Int. J. Cancer.

[58] Facciabene A., Peng X., Hagemann I.S., Balint K., Barchetti A., Wang L.P., Gimotty P.A., Gilks C.B., Lal P., Zhang L. (2011). Tumour hypoxia promotes tolerance and angiogenesis via CCL28 and T(reg) cells. Nature.

[59] Redjimi N., Raffin C., Raimbaud I., Pignon P., Matsuzaki J., Odunsi K., Valmori D., Ayyoub M. (2012). CXCR3+ T regulatory cells selectively accumulate in human ovarian carcinomas to limit type I immunity. Cancer Res..

[60] Mhawech-Fauceglia P., Wang D., Ali L., Lele S., Huba M.A., Liu S., Odunsi K. (2013). Intraepithelial T-cells and tumor-associated macrophages in ovarian cancer patients. Cancer Immun..

[61] Milne K., Köbel M., Kalloger S.E., Barnes R.O., Gao D., Gilks C.B., Watson P.H., Nelson B.H. (2009). Systematic analysis of immune infiltrates in high-grade serous ovarian cancer reveals CD20, FoxP3 and TIA-1 as positive prognostic factors. PLoS ONE.

[62] Wang R.F., Wang H.Y. (2017). Immune targets and neoantigens for cancer immunotherapy and precision medicine. Cell Res..

[63] Han L.Y., Fletcher M.S., Urbauer D.L., Mueller P., Landen C.N., Kamat A.A., Lin Y.G., Merritt W.M., Spannuth W.A., Deavers M.T. (2008). HLA class I antigen processing machinery component expression and intratumoral T-Cell infiltrate as independent prognostic markers in ovarian carcinoma. Clin. Cancer Res. Off. J. Am. Assoc. Cancer Res..

[64] Chalmers Z.R., Connelly C.F., Fabrizio D., Gay L., Ali S.M., Ennis R., Schrock A., Campbell B., Shlien A., Chmielecki J. (2017). Analysis of 100,000 human cancer genomes reveals the landscape of tumor mutational burden. Genome Med..

[65] Wick D.A., Webb J.R., Nielsen J.S., Martin S.D., Kroeger D.R., Milne K., Castellarin M., Twumasi-Boateng K., Watson P.H., Holt R.A. (2014). Surveillance of the tumor mutanome by T-cells during progression from primary to recurrent ovarian cancer. Clin. Cancer Res. Off. J. Am. Assoc. Cancer Res..

[66] Bobisse S., Genolet R., Roberti A., Tanyi J.L., Racle J., Stevenson B.J., Iseli C., Michel A., Le Bitoux M.A., Guillaume P. (2018). Sensitive and frequent identification of high avidity neo-epitope specific CD8 + T-cells in immunotherapy-naive ovarian cancer. Nat. Commun..

[67] Odunsi K., Jungbluth A.A., Stockert E., Qian F., Gnjatic S., Tammela J., Intengan M., Beck A., Keitz B., Santiago D. (2003). NY-ESO-1 and LAGE-1 cancer-testis antigens are potential targets for immunotherapy in epithelial ovarian cancer. Cancer Res..

[68] Matsuzaki J., Qian F., Luescher I., Lele S., Ritter G., Shrikant P.A., Gnjatic S., Old L.J., Odunsi K. (2008). Recognition of naturally processed and ovarian cancer reactive CD8+ T-cell epitopes within a promiscuous HLA class II T-helper region of NY-ESO-1. Cancer Immunol. Immunother..

[69] Odunsi K., Qian F., Matsuzaki J., Mhawech-Fauceglia P., Andrews C., Hoffman E.W., Pan L., Ritter G., Villella J., Thomas B. (2007). Vaccination with an NY-ESO-1 peptide of HLA class I/II specificities induces integrated humoral and T-cell responses in ovarian cancer. Proc. Natl. Acad. Sci. USA.

[70] Westergaard M.C.W., Andersen R., Chong C., Kjeldsen J.W., Pedersen M., Friese C., Hasselager T., Lajer H., Coukos G., Bassani-Sternberg M. (2019). Tumour-reactive T-cell subsets in the microenvironment of ovarian cancer. Br. J. Cancer.

[71] Schuster H., Peper J.K., Bösmüller H.C., Röhle K., Backert L., Bilich T., Ney B., Löffler M.W., Kowalewski D.J., Trautwein N. (2017). The immunopeptidomic landscape of ovarian carcinomas. Proc. Natl. Acad. Sci. USA.

[72] Sharonov G.V., Serebrovskaya E.O., Yuzhakova D.V., Britanova O.V., Chudakov D.M. (2020). B cells, plasma cells and antibody repertoires in the tumour microenvironment. Nat. Rev. Immunol..

[73] Gupta P., Chen C., Chaluvally-Raghavan P., Pradeep S. (2019). B Cells as an Immune-Regulatory Signature in Ovarian Cancer. Cancers.

[74] Santoiemma P.P., Reyes C., Wang L.P., McLane M.W., Feldman M.D., Tanyi J.L., Powell D.J. (2016). Systematic evaluation of multiple immune markers reveals prognostic factors in ovarian cancer. Gynecol. Oncol..

[75] Montfort A., Pearce O., Maniati E., Vincent B.G., Bixby L., Böhm S., Dowe T., Wilkes E.H., Chakravarty P., Thompson R. (2017). A Strong B-cell Response Is Part of the Immune Landscape in Human High-Grade Serous Ovarian Metastases. Clin. Cancer Res. Off. J. Am. Assoc. Cancer Res..

[76] Lundgren S., Berntsson J., Nodin B., Micke P., Jirström K. (2016). Prognostic impact of tumour-associated B cells and plasma cells in epithelial ovarian cancer. J. Ovarian Res..

[77] Yang C., Lee H., Jove V., Deng J., Zhang W., Liu X., Forman S., Dellinger T.H., Wakabayashi M., Yu H. (2013). Prognostic significance of B-cells and pSTAT3 in patients with ovarian cancer. PLoS ONE.

[78] Dong H.P., Elstrand M.B., Holth A., Silins I., Berner A., Trope C.G., Davidson B., Risberg B. (2006). NK- and B-cell infiltration correlates with worse outcome in metastatic ovarian carcinoma. Am. J. Clin. Pathol..

[79] He Y., Qian H., Liu Y., Duan L., Li Y., Shi G. (2014). The roles of regulatory B cells in cancer. J. Immunol. Res..

[80] Lindner S., Dahlke K., Sontheimer K., Hagn M., Kaltenmeier C., Barth T.F.E., Beyer T., Reister F., Fabricius D., Lotfi R. (2013). Interleukin 21-induced granzyme B-expressing B cells infiltrate tumors and regulate T-cells. Cancer Res..

[81] Morvan M.G., Lanier L.L. (2016). NK cells and cancer: You can teach innate cells new tricks. Nat. Rev. Cancer.

[82] Lai P., Rabinowich H., Crowley-Nowick P.A., Bell M.C., Mantovani G., Whiteside T.L. (1996). Alterations in expression and function of signal-transducing proteins in tumor-associated T and natural killer cells in patients with ovarian carcinoma. Clin. Cancer Res. Off. J. Am. Assoc. Cancer Res..

[83] Yunusova N.V., Stakheyeva M.N., Molchanov S.V., Afanas’ev S.G., Tsydenova A.A., Kolomiets L.A., Cherdyntseva N.V. (2018). Functional activity of natural killer cells in biological fluids in patients with colorectal and ovarian cancers. Cent. Eur. J. Immunol..

[84] Pesce S., Tabellini G., Cantoni C., Patrizi O., Coltrini D., Rampinelli F., Matta J., Vivier E., Moretta A., Parolini S. (2015). B7-H6-mediated downregulation of NKp30 in NK cells contributes to ovarian carcinoma immune escape. Oncoimmunology.

[85] Carlsten M., Norell H., Bryceson Y.T., Poschke I., Schedvins K., Ljunggren H.G., Kiessling R., Malmberg K.J. (2009). Primary human tumor cells expressing CD155 impair tumor targeting by down-regulating DNAM-1 on NK cells. J. Immunol..

[86] Gubbels J.A.A., Felder M., Horibata S., Belisle J.A., Kapur A., Holden H., Petrie S., Migneault M., Rancourt C., Connor J.P. (2010). MUC16 provides immune protection by inhibiting synapse formation between NK and ovarian tumor cells. Mol. Cancer.

[87] Li K., Mandai M., Hamanishi J., Matsumura N., Suzuki A., Yagi H., Yamaguchi K., Baba T., Fujii S., Konishi I. (2009). Clinical significance of the NKG2D ligands, MICA/B and ULBP2 in ovarian cancer: High expression of ULBP2 is an indicator of poor prognosis. Cancer Immunol. Immunother..

[88] Bösmüller H.C., Wagner P., Peper J.K., Schuster H., Pham D.L., Greif K., Beschorner C., Rammensee H.G., Stevanović S., Fend F. (2016). Combined Immunoscore of CD103 and CD3 Identifies Long-Term Survivors in High-Grade Serous Ovarian Cancer. Int. J. Gynecol. Cancer Off. J. Int. Gynecol. Cancer Soc..

[89] Hoogstad-van Evert J.S., Maas R.J., van der Meer J., Cany J., van der Steen S., Jansen J.H., Miller J.S., Bekkers R., Hobo W., Massuger L. (2018). Peritoneal NK cells are responsive to IL-15 and percentages are correlated with outcome in advanced ovarian cancer patients. Oncotarget.

[90] Garzetti G.G., Cignitti M., Ciavattini A., Fabris N., Romanini C. (1993). Natural killer cell activity and progression-free survival in ovarian cancer. Gynecol. Obstet. Investig..

[91] Hoogstad-van Evert J.S., Bekkers R., Ottevanger N., Jansen J.H., Massuger L., Dolstra H. (2020). Harnessing natural killer cells for the treatment of ovarian cancer. Gynecol. Oncol..

[92] Hoogstad-van Evert J.S., Cany J., van den Brand D., Oudenampsen M., Brock R., Torensma R., Bekkers R.L., Jansen J.H., Massuger L.F., Dolstra H. (2017). Umbilical cord blood CD34+ progenitor-derived NK cells efficiently kill ovarian cancer spheroids and intraperitoneal tumors in NOD/SCID/IL2Rgnull mice. Oncoimmunology.

[93] Haas L., Obenauf A.C. (2019). Allies or Enemies-The Multifaceted Role of Myeloid Cells in the Tumor Microenvironment. Front. Immunol..

[94] Okabe Y., Medzhitov R. (2014). Tissue-specific signals control reversible program of localization and functional polarization of macrophages. Cell.

[95] Gupta V., Yull F., Khabele D. (2018). Bipolar Tumor-Associated Macrophages in Ovarian Cancer as Targets for Therapy. Cancers.

[96] Reinartz S., Schumann T., Finkernagel F., Wortmann A., Jansen J.M., Meissner W., Krause M., Schwörer A.M., Wagner U., Müller-Brüsselbach S. (2014). Mixed-polarization phenotype of ascites-associated macrophages in human ovarian carcinoma: Correlation of CD163 expression, cytokine levels and early relapse. Int. J. Cancer.

[97] Colvin E.K. (2014). Tumor-associated macrophages contribute to tumor progression in ovarian cancer. Front. Oncol..

[98] Kawamura K., Komohara Y., Takaishi K., Katabuchi H., Takeya M. (2009). Detection of M2 macrophages and colony-stimulating factor 1 expression in serous and mucinous ovarian epithelial tumors. Pathol. Int..

[99] Lan C., Huang X., Lin S., Huang H., Cai Q., Wan T., Lu J., Liu J. (2013). Expression of M2-polarized macrophages is associated with poor prognosis for advanced epithelial ovarian cancer. Technol. Cancer Res. Treat..

[100] Schutyser E., Struyf S., Proost P., Opdenakker G., Laureys G., Verhasselt B., Peperstraete L., Van de Putte I., Saccani A., Allavena P. (2002). Identification of biologically active chemokine isoforms from ascitic fluid and elevated levels of CCL18/pulmonary and activation-regulated chemokine in ovarian carcinoma. J. Biol. Chem..

[101] Sica G.L., Choi I.H., Zhu G., Tamada K., Wang S.D., Tamura H., Chapoval A.I., Flies D.B., Bajorath J., Chen L. (2003). B7-H4, a molecule of the B7 family, negatively regulates T-cell immunity. Immunity.

[102] Zhou J., Li X., Wu X., Zhang T., Zhu Q., Wang X., Wang H., Wang K., Lin Y., Wang X. (2018). Exosomes Released from Tumor-Associated Macrophages Transfer miRNAs That Induce a Treg/Th17 Cell Imbalance in Epithelial Ovarian Cancer. Cancer Immunol. Res..

[103] Schumann T., Adhikary T., Wortmann A., Finkernagel F., Lieber S., Schnitzer E., Legrand N., Schober Y., Nockher W.A., Toth P.M. (2015). Deregulation of PPARβ/δ target genes in tumor-associated macrophages by fatty acid ligands in the ovarian cancer microenvironment. Oncotarget.

[104] El-Arabey A.A., Denizli M., Kanlikilicer P., Bayraktar R., Ivan C., Rashed M., Kabil N., Ozpolat B., Calin G.A., Salama S.A. (2020). GATA3 as a master regulator for interactions of tumor-associated macrophages with high-grade serous ovarian carcinoma. Cell. Signal..

[105] Yin M., Li X., Tan S., Zhou H.J., Ji W., Bellone S., Xu X., Zhang H., Santin A.D., Lou G. (2016). Tumor-associated macrophages drive spheroid formation during early transcoelomic metastasis of ovarian cancer. J. Clin. Investig..

[106] Yin M., Shen J., Yu S., Fei J., Zhu X., Zhao J., Zhai L., Sadhukhan A., Zhou J. (2019). Tumor-Associated Macrophages (TAMs): A Critical Activator In Ovarian Cancer Metastasis. OncoTargets Ther..

[107] Zeng X.Y., Xie H., Yuan J., Jiang X.Y., Yong J.H., Zeng D., Dou Y.Y., Xiao S.S. (2019). M2-like tumor-associated macrophages-secreted EGF promotes epithelial ovarian cancer metastasis via activating EGFR-ERK signaling and suppressing lncRNA LIMT expression. Cancer Biol. Ther..

[108] Etzerodt A., Moulin M., Doktor T.K., Delfini M., Mossadegh-Keller N., Bajenoff M., Sieweke M.H., Moestrup S.K., Auphan-Anezin N., Lawrence T. (2020). Tissue-resident macrophages in omentum promote metastatic spread of ovarian cancer. J. Exp. Med..

[109] He Y., Zhang M., Wu X., Sun X., Xu T., He Q., Di W. (2013). High MUC2 expression in ovarian cancer is inversely associated with the M1/M2 ratio of tumor-associated macrophages and patient survival time. PLoS ONE.

[110] Zhang M., He Y., Sun X., Li Q., Wang W., Zhao A., Di W. (2014). A high M1/M2 ratio of tumor-associated macrophages is associated with extended survival in ovarian cancer patients. J. Ovarian Res..

[111] Adhikary T., Wortmann A., Finkernagel F., Lieber S., Nist A., Stiewe T., Wagner U., Müller-Brüsselbach S., Reinartz S., Müller R. (2017). Interferon signaling in ascites-associated macrophages is linked to a favorable clinical outcome in a subgroup of ovarian carcinoma patients. BMC Genom..

[112] Luo C., Shibata K., Suzuki S., Kajiyama H., Senga T., Koya Y., Daimon M., Yamashita M., Kikkawa F. (2014). GPC3 expression in mouse ovarian cancer induces GPC3-specific T-cell-mediated immune response through M1 macrophages and suppresses tumor growth. Oncol. Rep..

[113] Dangaj D., Bruand M., Grimm A.J., Ronet C., Barras D., Duttagupta P.A., Lanitis E., Duraiswamy J., Tanyi J.L., Benencia F. (2019). Cooperation between Constitutive and Inducible Chemokines Enables T-cell Engraftment and Immune Attack in Solid Tumors. Cancer Cell.

[114] Gabrilovich D.I. (2017). Myeloid-Derived Suppressor Cells. Cancer Immunol. Res..

[115] Cui T.X., Kryczek I., Zhao L., Zhao E., Kuick R., Roh M.H., Vatan L., Szeliga W., Mao Y., Thomas D.G. (2013). Myeloid-derived suppressor cells enhance stemness of cancer cells by inducing microRNA101 and suppressing the corepressor CtBP2. Immunity.

[116] Okła K., Czerwonka A., Wawruszak A., Bobiński M., Bilska M., Tarkowski R., Bednarek W., Wertel I., Kotarski J. (2019). Clinical Relevance and Immunosuppressive Pattern of Circulating and Infiltrating Subsets of Myeloid-Derived Suppressor Cells (MDSCs) in Epithelial Ovarian Cancer. Front. Immunol..

[117] Wu L., Deng Z., Peng Y., Han L., Liu J., Wang L., Li B., Zhao J., Jiao S., Wei H. (2017). Ascites-derived IL-6 and IL-10 synergistically expand CD14+HLA-DR-/low myeloid-derived suppressor cells in ovarian cancer patients. Oncotarget.

[118] Coosemans A., Baert T., Ceusters J., Busschaert P., Landolfo C., Verschuere T., Van Rompuy A.S., Vanderstichele A., Froyman W., Neven P. (2019). Myeloid-derived suppressor cells at diagnosis may discriminate between benign and malignant ovarian tumors. Int. J. Gynecol. Cancer Off. J. Int. Gynecol. Cancer Soc..

[119] Taki M., Abiko K., Baba T., Hamanishi J., Yamaguchi K., Murakami R., Yamanoi K., Horikawa N., Hosoe Y., Nakamura E. (2018). Snail promotes ovarian cancer progression by recruiting myeloid-derived suppressor cells via CXCR2 ligand upregulation. Nat. Commun..

[120] Obermajer N., Muthuswamy R., Odunsi K., Edwards R.P., Kalinski P. (2011). PGE(2)-induced CXCL12 production and CXCR4 expression controls the accumulation of human MDSCs in ovarian cancer environment. Cancer Res..

[121] Horikawa N., Abiko K., Matsumura N., Hamanishi J., Baba T., Yamaguchi K., Yoshioka Y., Koshiyama M., Konishi I. (2017). Expression of Vascular Endothelial Growth Factor in Ovarian Cancer Inhibits Tumor Immunity through the Accumulation of Myeloid-Derived Suppressor Cells. Clin. Cancer Res. Off. J. Am. Assoc. Cancer Res..

[122] Horikawa N., Abiko K., Matsumura N., Baba T., Hamanishi J., Yamaguchi K., Murakami R., Taki M., Ukita M., Hosoe Y. (2020). Anti-VEGF therapy resistance in ovarian cancer is caused by GM-CSF-induced myeloid-derived suppressor cell recruitment. Br. J. Cancer.

[123] Chen X., Shang W., Xu R., Wu M., Zhang X., Huang P., Wang F., Pan S. (2019). Distribution and functions of γδ T-cells infiltrated in the ovarian cancer microenvironment. J. Transl. Med..

[124] Rei M., Gonçalves-Sousa N., Lança T., Thompson R.G., Mensurado S., Balkwill F.R., Kulbe H., Pennington D.J., Silva-Santos B. (2014). Murine CD27(-) Vγ6(+) γδ T-cells producing IL-17A promote ovarian cancer growth via mobilization of protumor small peritoneal macrophages. Proc. Natl. Acad. Sci. USA.

[125] Chen X., Zhang X., Xu R., Shang W., Ming W., Wang F., Wang J. (2020). Implication of IL-17 producing αβT and γδT cells in patients with ovarian cancer. Hum. Immunol..

[126] Huang Q.T., Zhou L., Zeng W.J., Ma Q.Q., Wang W., Zhong M., Yu Y.H. (2017). Prognostic Significance of Neutrophil-to-Lymphocyte Ratio in Ovarian Cancer: A Systematic Review and Meta-Analysis of Observational Studies. Cell. Physiol. Biochem..

[127] Zhou Q., Hong L., Zuo M.Z., He Z. (2017). Prognostic significance of neutrophil to lymphocyte ratio in ovarian cancer: Evidence from 4,910 patients. Oncotarget.

[128] Lee W., Ko S.Y., Mohamed M.S., Kenny H.A., Lengyel E., Naora H. (2019). Neutrophils facilitate ovarian cancer premetastatic niche formation in the omentum. J. Exp. Med..

[129] Singel K.L., Emmons T.R., Khan A.N.H., Mayor P.C., Shen S., Wong J.T., Morrell K., Eng K.H., Mark J., Bankert R.B. (2019). Mature neutrophils suppress T-cell immunity in ovarian cancer microenvironment. JCI Insight.

[130] Shang A., Wang W., Gu C., Chen C., Zeng B., Yang Y., Ji P., Sun J., Wu J., Lu W. (2019). Long non-coding RNA HOTTIP enhances IL-6 expression to potentiate immune escape of ovarian cancer cells by upregulating the expression of PD-L1 in neutrophils. J. Exp. Clin. Cancer Res..

[131] Tudrej P., Kujawa K.A., Cortez A.J., Lisowska K.M. (2019). Characteristics of in Vivo Model Systems for Ovarian Cancer Studies. Diagnostics.

[132] Perets R., Wyant G.A., Muto K.W., Bijron J.G., Poole B.B., Chin K.T., Chen J.Y.H., Ohman A.W., Stepule C.D., Kwak S. (2013). Transformation of the fallopian tube secretory epithelium leads to high-grade serous ovarian cancer in Brca;Tp53;Pten models. Cancer Cell.

[133] Wieser V., Gaugg I., Fleischer M., Shivalingaiah G., Wenzel S., Sprung S., Lax S.F., Zeimet A.G., Fiegl H., Marth C. (2018). BRCA1/2 and TP53 mutation status associates with PD-1 and PD-L1 expression in ovarian cancer. Oncotarget.

[134] Son D.S., Kabir S.M., Dong Y.L., Lee E., Adunyah S.E. (2012). Inhibitory effect of tumor suppressor p53 on proinflammatory chemokine expression in ovarian cancer cells by reducing proteasomal degradation of IκB. PLoS ONE.

[135] Maniati E., Berlato C., Gopinathan G., Heath O., Kotantaki P., Lakhani A., McDermott J., Pegrum C., Delaine-Smith R.M., Pearce O.M.T. (2020). Mouse Ovarian Cancer Models Recapitulate the Human Tumor Microenvironment and Patient Response to Treatment. Cell Rep..

[136] Magnotti E., Marasco W.A. (2018). The latest animal models of ovarian cancer for novel drug discovery. Expert Opin. Drug Discov..

[137] Gitto S.B., Kim H., Rafail S., Omran D.K., Medvedev S., Kinose Y., Rodriguez-Garcia A., Flowers A.J., Xu H., Schwartz L.E. (2020). An autologous humanized patient-derived-xenograft platform to evaluate immunotherapy in ovarian cancer. Gynecol. Oncol..

[138] Roby K.F., Taylor C.C., Sweetwood J.P., Cheng Y., Pace J.L., Tawfik O., Persons D.L., Smith P.G., Terranova P.F. (2000). Development of a syngeneic mouse model for events related to ovarian cancer. Carcinogenesis.

[139] Wei H., Zhao L., Li W., Fan K., Qian W., Hou S., Wang H., Dai M., Hellstrom I., Hellstrom K.E. (2013). Combinatorial PD-1 blockade and CD137 activation has therapeutic efficacy in murine cancer models and synergizes with cisplatin. PLoS ONE.

[140] Guo Z., Wang X., Cheng D., Xia Z., Luan M., Zhang S. (2014). PD-1 blockade and OX40 triggering synergistically protects against tumor growth in a murine model of ovarian cancer. PLoS ONE.

[141] Chiang C.L.-L., Kandalaft L.E., Tanyi J., Hagemann A.R., Motz G.T., Svoronos N., Montone K., Mantia-Smaldone G.M., Smith L., Nisenbaum H.L. (2013). A dendritic cell vaccine pulsed with autologous hypochlorous acid-oxidized ovarian cancer lysate primes effective broad antitumor immunity: From bench to bedside. Clin. Cancer Res. Off. J. Am. Assoc. Cancer Res..

[142] Leinster D.A., Kulbe H., Everitt G., Thompson R., Perretti M., Gavins F.N.E., Cooper D., Gould D., Ennis D.P., Lockley M. (2012). The peritoneal tumour microenvironment of high-grade serous ovarian cancer. J. Pathol..

[143] Cole A.J., Dwight T., Gill A.J., Dickson K.A., Zhu Y., Clarkson A., Gard G.B., Maidens J., Valmadre S., Clifton-Bligh R. (2016). Assessing mutant p53 in primary high-grade serous ovarian cancer using immunohistochemistry and massively parallel sequencing. Sci. Rep..

[144] Walton J., Blagih J., Ennis D., Leung E., Dowson S., Farquharson M., Tookman L.A., Orange C., Athineos D., Mason S. (2016). CRISPR/Cas9-Mediated Trp53 and Brca2 Knockout to Generate Improved Murine Models of Ovarian High-Grade Serous Carcinoma. Cancer Res..

[145] Pardoll D.M. (2012). The blockade of immune checkpoints in cancer immunotherapy. Nat. Rev. Cancer.

[146] Abiko K., Mandai M., Hamanishi J., Yoshioka Y., Matsumura N., Baba T., Yamaguchi K., Murakami R., Yamamoto A., Kharma B. (2013). PD-L1 on tumor cells is induced in ascites and promotes peritoneal dissemination of ovarian cancer through CTL dysfunction. Clin. Cancer Res. Off. J. Am. Assoc. Cancer Res..

[147] Huang R.Y., Francois A., McGray A.R., Miliotto A., Odunsi K. (2017). Compensatory upregulation of PD-1, LAG-3, and CTLA-4 limits the efficacy of single-agent checkpoint blockade in metastatic ovarian cancer. Oncoimmunology.

[148] Duraiswamy J., Kaluza K.M., Freeman G.J., Coukos G. (2013). Dual blockade of PD-1 and CTLA-4 combined with tumor vaccine effectively restores T-cell rejection function in tumors. Cancer Res..

[149] Lu L., Xu X., Zhang B., Zhang R., Ji H., Wang X. (2014). Combined PD-1 blockade and GITR triggering induce a potent antitumor immunity in murine cancer models and synergizes with chemotherapeutic drugs. J. Transl. Med..

[150] Guo Z., Cheng D., Xia Z., Luan M., Wu L., Wang G., Zhang S. (2013). Combined TIM-3 blockade and CD137 activation affords the long-term protection in a murine model of ovarian cancer. J. Transl. Med..

[151] Peng J., Hamanishi J., Matsumura N., Abiko K., Murat K., Baba T., Yamaguchi K., Horikawa N., Hosoe Y., Murphy S.K. (2015). Chemotherapy Induces Programmed Cell Death-Ligand 1 Overexpression via the Nuclear Factor-κB to Foster an Immunosuppressive Tumor Microenvironment in Ovarian Cancer. Cancer Res..

[152] Guo Z., Wang H., Meng F., Li J., Zhang S. (2015). Combined Trabectedin and anti-PD1 antibody produces a synergistic antitumor effect in a murine model of ovarian cancer. J. Transl. Med..

[153] Zhu X., Xu J., Cai H., Lang J. (2018). Carboplatin and programmed death-ligand 1 blockade synergistically produce a similar antitumor effect to carboplatin alone in murine ID8 ovarian cancer model. J. Obstet. Gynaecol. Res..

[154] Ghaffari A., Peterson N., Khalaj K., Vitkin N., Robinson A., Francis J.A., Koti M. (2018). STING agonist therapy in combination with PD-1 immune checkpoint blockade enhances response to carboplatin chemotherapy in high-grade serous ovarian cancer. Br. J. Cancer.

[155] Wang L., Amoozgar Z., Huang J., Saleh M.H., Xing D., Orsulic S., Goldberg M.S. (2015). Decitabine Enhances Lymphocyte Migration and Function and Synergizes with CTLA-4 Blockade in a Murine Ovarian Cancer Model. Cancer Immunol. Res..

[156] Higuchi T., Flies D.B., Marjon N.A., Mantia-Smaldone G., Ronner L., Gimotty P.A., Adams S.F. (2015). CTLA-4 Blockade Synergizes Therapeutically with PARP Inhibition in BRCA1-Deficient Ovarian Cancer. Cancer Immunol. Res..

[157] Wang Z., Sun K., Xiao Y., Feng B., Mikule K., Ma X., Feng N., Vellano C.P., Federico L., Marszalek J.R. (2019). Niraparib activates interferon signaling and potentiates anti-PD-1 antibody efficacy in tumor models. Sci. Rep..

[158] Nesbeth Y., Scarlett U., Cubillos-Ruiz J., Martinez D., Engle X., Turk M.J., Conejo-Garcia J.R. (2009). CCL5-mediated endogenous antitumor immunity elicited by adoptively transferred lymphocytes and dendritic cell depletion. Cancer Res..

[159] Chruściel E., Urban-Wójciuk Z., Arcimowicz Ł., Kurkowiak M., Kowalski J., Gliwiński M., Marjański T., Rzyman W., Biernat W., Dziadziuszko R. (2020). Adoptive Cell Therapy-Harnessing Antigen-Specific T-cells to Target Solid Tumours. Cancers.

[160] Chekmasova A.A., Rao T.D., Nikhamin Y., Park K.J., Levine D.A., Spriggs D.R., Brentjens R.J. (2010). Successful eradication of established peritoneal ovarian tumors in SCID-Beige mice following adoptive transfer of T-cells genetically targeted to the MUC16 antigen. Clin. Cancer Res. Off. J. Am. Assoc. Cancer Res..

[161] Koneru M., Purdon T.J., Spriggs D., Koneru S., Brentjens R.J. (2015). IL-12 secreting tumor-targeted chimeric antigen receptor T-cells eradicate ovarian tumors in vivo. Oncoimmunology.

[162] Anderson K.G., Voillet V., Bates B.M., Chiu E.Y., Burnett M.G., Garcia N.M., Oda S.K., Morse C.B., Stromnes I.M., Drescher C.W. (2019). Engineered Adoptive T-cell Therapy Prolongs Survival in a Preclinical Model of Advanced-Stage Ovarian Cancer. Cancer Immunol. Res..

[163] Song D.G., Ye Q., Carpenito C., Poussin M., Wang L.P., Ji C., Figini M., June C.H., Coukos G., Powell D.J. (2011). In vivo persistence, tumor localization, and antitumor activity of CAR-engineered T-cells is enhanced by costimulatory signaling through CD137 (4-1BB). Cancer Res..

[164] Owens G.L., Sheard V.E., Kalaitsidou M., Blount D., Lad Y., Cheadle E.J., Edmondson R.J., Kooner G., Gilham D.E., Harrop R. (2018). Preclinical Assessment of CAR T-Cell Therapy Targeting the Tumor Antigen 5T4 in Ovarian Cancer. J. Immunother..

[165] Du H., Hirabayashi K., Ahn S., Kren N.P., Montgomery S.A., Wang X., Tiruthani K., Mirlekar B., Michaud D., Greene K. (2019). Antitumor Responses in the Absence of Toxicity in Solid Tumors by Targeting B7-H3 via Chimeric Antigen Receptor T-cells. Cancer Cell.

[166] Ang W.X., Li Z., Chi Z., Du S.H., Chen C., Tay J.C.K., Toh H.C., Connolly J.E., Xu X.H., Wang S. (2017). Intraperitoneal immunotherapy with T-cells stably and transiently expressing anti-EpCAM CAR in xenograft models of peritoneal carcinomatosis. Oncotarget.

[167] Whilding L.M., Parente-Pereira A.C., Zabinski T., Davies D.M., Petrovic R.M.G., Kao Y.V., Saxena S.A., Romain A., Costa-Guerra J.A., Violette S. (2017). Targeting of Aberrant αvβ6 Integrin Expression in Solid Tumors Using Chimeric Antigen Receptor-Engineered T-cells. Mol. Ther. J. Am. Soc. Gene Ther..

[168] Hong H., Brown C.E., Ostberg J.R., Priceman S.J., Chang W.C., Weng L., Lin P., Wakabayashi M.T., Jensen M.C., Forman S.J. (2016). L1 Cell Adhesion Molecule-Specific Chimeric Antigen Receptor-Redirected Human T-cells Exhibit Specific and Efficient Antitumor Activity against Human Ovarian Cancer in Mice. PLoS ONE.

[169] Wilson A.J., Saskowski J., Barham W., Khabele D., Yull F. (2015). Microenvironmental effects limit efficacy of thymoquinone treatment in a mouse model of ovarian cancer. Mol. Cancer.

[170] Moughon D.L., He H., Schokrpur S., Jiang Z.K., Yaqoob M., David J., Lin C., Iruela-Arispe M.L., Dorigo O., Wu L. (2015). Macrophage Blockade Using CSF1R Inhibitors Reverses the Vascular Leakage Underlying Malignant Ascites in Late-Stage Epithelial Ovarian Cancer. Cancer Res..

[171] Lyons Y.A., Pradeep S., Wu S.Y., Haemmerle M., Hansen J.M., Wagner M.J., Villar-Prados A., Nagaraja A.S., Dood R.L., Previs R.A. (2017). Macrophage depletion through colony stimulating factor 1 receptor pathway blockade overcomes adaptive resistance to anti-VEGF therapy. Oncotarget.

[172] Germano G., Frapolli R., Belgiovine C., Anselmo A., Pesce S., Liguori M., Erba E., Uboldi S., Zucchetti M., Pasqualini F. (2013). Role of macrophage targeting in the antitumor activity of trabectedin. Cancer Cell.

[173] Penn C.A., Yang K., Zong H., Lim J.Y., Cole A., Yang D., Baker J., Goonewardena S.N., Buckanovich R.J. (2018). Therapeutic Impact of Nanoparticle Therapy Targeting Tumor-Associated Macrophages. Mol. Cancer Ther..

[174] Pulaski H.L., Spahlinger G., Silva I.A., McLean K., Kueck A.S., Reynolds R.K., Coukos G., Conejo-Garcia J.R., Buckanovich R.J. (2009). Identifying alemtuzumab as an anti-myeloid cell antiangiogenic therapy for the treatment of ovarian cancer. J. Transl. Med..

[175] Kono Y., Kawakami S., Higuchi Y., Maruyama K., Yamashita F., Hashida M. (2014). Tumour-associated macrophages targeted transfection with NF-κB decoy/mannose-modified bubble lipoplexes inhibits tumour growth in tumour-bearing mice. J. Drug Target..

[176] Zhang F., Parayath N.N., Ene C.I., Stephan S.B., Koehne A.L., Coon M.E., Holland E.C., Stephan M.T. (2019). Genetic programming of macrophages to perform antitumor functions using targeted mRNA nanocarriers. Nat. Commun..

[177] Zhang Q., Li Y., Miao C., Wang Y., Xu Y., Dong R., Zhang Z., Griffin B.B., Yuan C., Yan S. (2018). Anti-angiogenesis effect of Neferine via regulating autophagy and polarization of tumor-associated macrophages in high-grade serous ovarian carcinoma. Cancer Lett..

[178] Zhu H., Bengsch F., Svoronos N., Rutkowski M.R., Bitler B.G., Allegrezza M.J., Yokoyama Y., Kossenkov A.V., Bradner J.E., Conejo-Garcia J.R. (2016). BET Bromodomain Inhibition Promotes Antitumor Immunity by Suppressing PD-L1 Expression. Cell Rep..

[179] Stone M.L., Chiappinelli K.B., Li H., Murphy L.M., Travers M.E., Topper M.J., Mathios D., Lim M., Shih I.M., Wang T.L. (2017). Epigenetic therapy activates type I interferon signaling in murine ovarian cancer to reduce immunosuppression and tumor burden. Proc. Natl. Acad. Sci. USA.

[180] Travers M., Brown S.M., Dunworth M., Holbert C.E., Wiehagen K.R., Bachman K.E., Foley J.R., Stone M.L., Baylin S.B., Casero R.A. (2019). DFMO and 5-Azacytidine Increase M1 Macrophages in the Tumor Microenvironment of Murine Ovarian Cancer. Cancer Res..

[181] Chang D.K., Peterson E., Sun J., Goudie C., Drapkin R.I., Liu J.F., Matulonis U., Zhu Q., Marasco W.A. (2016). Anti-CCR4 monoclonal antibody enhances antitumor immunity by modulating tumor-infiltrating Tregs in an ovarian cancer xenograft humanized mouse model. Oncoimmunology.

[182] Righi E., Kashiwagi S., Yuan J., Santosuosso M., Leblanc P., Ingraham R., Forbes B., Edelblute B., Collette B., Xing D. (2011). CXCL12/CXCR4 blockade induces multimodal antitumor effects that prolong survival in an immunocompetent mouse model of ovarian cancer. Cancer Res..

[183] Zeng Y., Li B., Liang Y., Reeves P.M., Qu X., Ran C., Liu Q., Callahan M.V., Sluder A.E., Gelfand J.A. (2019). Dual blockade of CXCL12-CXCR4 and PD-1-PD-L1 pathways prolongs survival of ovarian tumor-bearing mice by prevention of immunosuppression in the tumor microenvironment. FASEB J. Off. Publ. Fed. Am. Soc. Exp. Biol..

[184] Chu C.S., Kim S.H., June C.H., Coukos G. (2008). Immunotherapy opportunities in ovarian cancer. Expert Rev. Anticancer Ther..

[185] Mu J., Zou J.P., Yamamoto N., Tsutsui T., Tai X.G., Kobayashi M., Herrmann S., Fujiwara H., Hamaoka T. (1995). Administration of recombinant interleukin 12 prevents outgrowth of tumor cells metastasizing spontaneously to lung and lymph nodes. Cancer Res..

[186] Yu W.G., Yamamoto N., Takenaka H., Mu J., Tai X.G., Zou J.P., Ogawa M., Tsutsui T., Wijesuriya R., Yoshida R. (1996). Molecular mechanisms underlying IFN-gamma-mediated tumor growth inhibition induced during tumor immunotherapy with rIL-12. Int. Immunol..

[187] Simpson-Abelson M.R., Purohit V.S., Pang W.M., Iyer V., Odunsi K., Demmy T.L., Yokota S.J., Loyall J.L., Kelleher R.J., Balu-Iyer S. (2009). IL-12 delivered intratumorally by multilamellar liposomes reactivates memory T-cells in human tumor microenvironments. Clin. Immunol..

[188] Fewell J.G., Matar M.M., Rice J.S., Brunhoeber E., Slobodkin G., Pence C., Worker M., Lewis D.H., Anwer K. (2009). Treatment of disseminated ovarian cancer using nonviral interleukin-12 gene therapy delivered intraperitoneally. J. Gene Med..

[189] Sanches R., Kuiper M., Penault-Llorca F., Aunoble B., D’Incan C., Bignon Y.J. (2000). Antitumoral effect of interleukin-12-secreting fibroblasts in a mouse model of ovarian cancer: Implications for the use of ovarian cancer biopsy-derived fibroblasts as a vehicle for regional gene therapy. Cancer Gene Ther..

[190] Thomas E.D., Meza-Perez S., Bevis K.S., Randall T.D., Gillespie G.Y., Langford C., Alvarez R.D. (2016). IL-12 Expressing oncolytic herpes simplex virus promotes antitumor activity and immunologic control of metastatic ovarian cancer in mice. J. Ovarian Res..

[191] Gao J.Q., Kanagawa N., Motomura Y., Yanagawa T., Sugita T., Hatanaka Y., Tani Y., Mizuguchi H., Tsutsumi Y., Mayumi T. (2007). Cotransduction of CCL27 gene can improve the efficacy and safety of IL-12 gene therapy for cancer. Gene Ther..

[192] Bankert R.B., Balu-Iyer S.V., Odunsi K., Shultz L.D., Kelleher R.J., Barnas J.L., Simpson-Abelson M., Parsons R., Yokota S.J. (2011). Humanized mouse model of ovarian cancer recapitulates patient solid tumor progression, ascites formation, and metastasis. PLoS ONE.

[193] Burke F., East N., Upton C., Patel K., Balkwill F.R. (1997). Interferon gamma induces cell cycle arrest and apoptosis in a model of ovarian cancer: Enhancement of effect by batimastat. Eur. J. Cancer.

[194] Tedjarati S., Baker C.H., Apte S., Huang S., Wolf J.K., Killion J.J., Fidler I.J. (2002). Synergistic therapy of human ovarian carcinoma implanted orthotopically in nude mice by optimal biological dose of pegylated interferon alpha combined with paclitaxel. Clin. Cancer Res. Off. J. Am. Assoc. Cancer Res..

[195] Nakashima H., Miyake K., Clark C.R., Bekisz J., Finbloom J., Husain S.R., Baron S., Puri R.K., Zoon K.C. (2012). Potent antitumor effects of combination therapy with IFNs and monocytes in mouse models of established human ovarian and melanoma tumors. Cancer Immunol. Immunother..

[196] Abiko K., Matsumura N., Hamanishi J., Horikawa N., Murakami R., Yamaguchi K., Yoshioka Y., Baba T., Konishi I., Mandai M. (2015). IFN-γ from lymphocytes induces PD-L1 expression and promotes progression of ovarian cancer. Br. J. Cancer.

[197] Guo C., Manjili M.H., Subjeck J.R., Sarkar D., Fisher P.B., Wang X.Y. (2013). Therapeutic cancer vaccines: Past, present, and future. Adv. Cancer Res..

[198] Martin S.D., Brown S.D., Wick D.A., Nielsen J.S., Kroeger D.R., Twumasi-Boateng K., Holt R.A., Nelson B.H. (2016). Low Mutation Burden in Ovarian Cancer May Limit the Utility of Neoantigen-Targeted Vaccines. PLoS ONE.

[199] Budiu R.A., Elishaev E., Brozick J., Lee M., Edwards R.P., Kalinski P., Vlad A.M. (2013). Immunobiology of human mucin 1 in a preclinical ovarian tumor model. Oncogene.

[200] Chang M.C., Chen Y.L., Chiang Y.C., Chen T.C., Tang Y.C., Chen C.A., Sun W.Z., Cheng W.F. (2016). Mesothelin-specific cell-based vaccine generates antigen-specific immunity and potent antitumor effects by combining with IL-12 immunomodulator. Gene Ther..

[201] Li L., Goedegebuure S.P., Gillanders W.E. (2017). Preclinical and clinical development of neoantigen vaccines. Ann. Oncol. Off. J. Eur. Soc. Med. Oncol..

[202] Maeng H., Terabe M., Berzofsky J.A. (2018). Cancer vaccines: Translation from mice to human clinical trials. Curr. Opin. Immunol..

[203] Odunsi K. (2017). Immunotherapy in ovarian cancer. Ann. Oncol. Off. J. Eur. Soc. Med. Oncol..

[204] Matulonis U.A., Shapira-Frommer R., Santin A.D., Lisyanskaya A.S., Pignata S., Vergote I., Raspagliesi F., Sonke G.S., Birrer M., Provencher D.M. (2019). Antitumor activity and safety of pembrolizumab in patients with advanced recurrent ovarian cancer: Results from the phase II KEYNOTE-100 study. Ann. Oncol. Off. J. Eur. Soc. Med. Oncol..

[205] Disis M.L., Taylor M.H., Kelly K., Beck J.T., Gordon M., Moore K.M., Patel M.R., Chaves J., Park H., Mita A.C. (2019). Efficacy and Safety of Avelumab for Patients With Recurrent or Refractory Ovarian Cancer: Phase 1b Results From the JAVELIN Solid Tumor Trial. JAMA Oncol..

[206] Hamanishi J., Mandai M., Ikeda T., Minami M., Kawaguchi A., Murayama T., Kanai M., Mori Y., Matsumoto S., Chikuma S. (2015). Safety and Antitumor Activity of Anti-PD-1 Antibody, Nivolumab, in Patients With Platinum-Resistant Ovarian Cancer. J. Clin. Oncol. Off. J. Am. Soc. Clin. Oncol..

[207] Zamarin D., Burger R.A., Sill M.W., Powell D.J., Lankes H.A., Feldman M.D., Zivanovic O., Gunderson C., Ko E., Mathews C. (2020). Randomized Phase II Trial of Nivolumab Versus Nivolumab and Ipilimumab for Recurrent or Persistent Ovarian Cancer: An NRG Oncology Study. J. Clin. Oncol. Off. J. Am. Soc. Clin. Oncol..

[208] Brahmer J.R., Tykodi S.S., Chow L.Q.M., Hwu W.J., Topalian S.L., Hwu P., Drake C.G., Camacho L.H., Kauh J., Odunsi K. (2012). Safety and activity of anti-PD-L1 antibody in patients with advanced cancer. N. Engl. J. Med..

[209] Hodi F.S., Butler M., Oble D.A., Seiden M.V., Haluska F.G., Kruse A., Macrae S., Nelson M., Canning C., Lowy I. (2008). Immunologic and clinical effects of antibody blockade of cytotoxic T lymphocyte-associated antigen 4 in previously vaccinated cancer patients. Proc. Natl. Acad. Sci. USA.

[210] Fantini M., David J.M., Saric O., Dubeykovskiy A., Cui Y., Mavroukakis S.A., Bristol A., Annunziata C.M., Tsang K.Y., Arlen P.M. (2017). Preclinical Characterization of a Novel Monoclonal Antibody NEO-201 for the Treatment of Human Carcinomas. Front. Immunol..

[211] Fantini M., David J.M., Annunziata C.M., Morelli M.P., Arlen P.M., Tsang K.Y. (2020). The Monoclonal Antibody NEO-201 Enhances Natural Killer Cell Cytotoxicity against Tumor Cells through Blockade of the Inhibitory CEACAM5/CEACAM1 Immune Checkpoint Pathway. Cancer Biother. Radiopharm..

[212] Moore K., Colombo N., Scambia G., Kim B.G., Oaknin A., Friedlander M., Lisyanskaya A., Floquet A., Leary A., Sonke G.S. (2018). Maintenance Olaparib in Patients with Newly Diagnosed Advanced Ovarian Cancer. N. Engl. J. Med..

[213] Mirza M.R., Monk B.J., Herrstedt J., Oza A.M., Mahner S., Redondo A., Fabbro M., Ledermann J.A., Lorusso D., Vergote I. (2016). Niraparib Maintenance Therapy in Platinum-Sensitive, Recurrent Ovarian Cancer. N. Engl. J. Med..

[214] Ledermann J., Harter P., Gourley C., Friedlander M., Vergote I., Rustin G., Scott C., Meier W., Shapira-Frommer R., Safra T. (2012). Olaparib maintenance therapy in platinum-sensitive relapsed ovarian cancer. N. Engl. J. Med..

[215] González-Martín A., Pothuri B., Vergote I., DePont Christensen R., Graybill W., Mirza M.R., McCormick C., Lorusso D., Hoskins P., Freyer G. (2019). Niraparib in Patients with Newly Diagnosed Advanced Ovarian Cancer. N. Engl. J. Med..

[216] Coleman R.L., Oza A.M., Lorusso D., Aghajanian C., Oaknin A., Dean A., Colombo N., Weberpals J.I., Clamp A., Scambia G. (2017). Rucaparib maintenance treatment for recurrent ovarian carcinoma after response to platinum therapy (ARIEL3): A randomised, double-blind, placebo-controlled, phase 3 trial. Lancet Lond. Engl..

[217] Johnston P.A., Sen M., Hua Y., Camarco D., Shun T.Y., Lazo J.S., Grandis J.R. (2011). Cancer Genome Atlas Research Network Integrated genomic analyses of ovarian carcinoma. Nature.

[218] Lord C.J., Ashworth A. (2017). PARP inhibitors: Synthetic lethality in the clinic. Science.

[219] Strickland K.C., Howitt B.E., Shukla S.A., Rodig S., Ritterhouse L.L., Liu J.F., Garber J.E., Chowdhury D., Wu C.J., D’Andrea A.D. (2016). Association and prognostic significance of BRCA1/2-mutation status with neoantigen load, number of tumor-infiltrating lymphocytes and expression of PD-1/PD-L1 in high grade serous ovarian cancer. Oncotarget.

[220] Stewart R.A., Pilié P.G., Yap T.A. (2018). Development of PARP and Immune-Checkpoint Inhibitor Combinations. Cancer Res..

[221] Drew Y., de Jonge M., Hong S.H., Park Y.H., Wolfer A., Brown J., Ferguson M., Gore M.E., Alvarez R.H., Gresty C. (2018). An open-label, phase II basket study of olaparib and durvalumab (MEDIOLA): Results in germline BRCA-mutated (gBRCAm) platinum-sensitive relapsed (PSR) ovarian cancer (OC). Gynecol. Oncol..

[222] Lee J.M., Cimino-Mathews A., Peer C.J., Zimmer A., Lipkowitz S., Annunziata C.M., Cao L., Harrell M.I., Swisher E.M., Houston N. (2017). Safety and Clinical Activity of the Programmed Death-Ligand 1 Inhibitor Durvalumab in Combination With Poly (ADP-Ribose) Polymerase Inhibitor Olaparib or Vascular Endothelial Growth Factor Receptor 1-3 Inhibitor Cediranib in Women’s Cancers: A Dose-Escalation, Phase I Study. J. Clin. Oncol. Off. J. Am. Soc. Clin. Oncol..

[223] Konstantinopoulos P.A., Vinayak S. (2019). BRCA Mutations and Homologous Recombination Repair Deficiency in Treatment with Niraparib Combined with Pembrolizumab-Reply. JAMA Oncol..

[224] Huang Y., Kim B.Y.S., Chan C.K., Hahn S.M., Weissman I.L., Jiang W. (2018). Improving immune-vascular crosstalk for cancer immunotherapy. Nat. Rev. Immunol..

[225] Zimmer A.S., Nichols E., Cimino-Mathews A., Peer C., Cao L., Lee M.J., Kohn E.C., Annunziata C.M., Lipkowitz S., Trepel J.B. (2019). A phase I study of the PD-L1 inhibitor, durvalumab, in combination with a PARP inhibitor, olaparib, and a VEGFR1-3 inhibitor, cediranib, in recurrent women’s cancers with biomarker analyses. J. Immunother. Cancer.

[226] Cheung A., Bax H.J., Josephs D.H., Ilieva K.M., Pellizzari G., Opzoomer J., Bloomfield J., Fittall M., Grigoriadis A., Figini M. (2016). Targeting folate receptor alpha for cancer treatment. Oncotarget.

[227] Brown T.A., Byrd K., Vreeland T.J., Clifton G.T., Jackson D.O., Hale D.F., Herbert G.S., Myers J.W., Greene J.M., Berry J.S. (2019). Final analysis of a phase I/IIa trial of the folate-binding protein-derived E39 peptide vaccine to prevent recurrence in ovarian and endometrial cancer patients. Cancer Med..

[228] Kalli K.R., Block M.S., Kasi P.M., Erskine C.L., Hobday T.J., Dietz A., Padley D., Gustafson M.P., Shreeder B., Puglisi-Knutson D. (2018). Folate Receptor Alpha Peptide Vaccine Generates Immunity in Breast and Ovarian Cancer Patients. Clin. Cancer Res. Off. J. Am. Assoc. Cancer Res..

[229] Moore K.N., O’Malley D.M., Vergote I., Martin L.P., Gonzalez-Martin A., Malek K., Birrer M.J. (2018). Safety and activity findings from a phase 1b escalation study of mirvetuximab soravtansine, a folate receptor alpha (FRα)-targeting antibody-drug conjugate (ADC), in combination with carboplatin in patients with platinum-sensitive ovarian cancer. Gynecol. Oncol..

[230] Moore K.N., Martin L.P., O’Malley D.M., Matulonis U.A., Konner J.A., Perez R.P., Bauer T.M., Ruiz-Soto R., Birrer M.J. (2017). Safety and Activity of Mirvetuximab Soravtansine (IMGN853), a Folate Receptor Alpha-Targeting Antibody-Drug Conjugate, in Platinum-Resistant Ovarian, Fallopian Tube, or Primary Peritoneal Cancer: A Phase I Expansion Study. J. Clin. Oncol. Off. J. Am. Soc. Clin. Oncol..

[231] Leffers N., Vermeij R., Hoogeboom B.N., Schulze U.R., Wolf R., Hamming I.E., van der Zee A.G., Melief K.J., van der Burg S.H., Daemen T. (2012). Long-term clinical and immunological effects of p53-SLP^®^ vaccine in patients with ovarian cancer. Int. J. Cancer.

[232] Vermeij R., Leffers N., Hoogeboom B.N., Hamming I.L.E., Wolf R., Reyners A.K.L., Molmans B.H.W., Hollema H., Bart J., Drijfhout J.W. (2012). Potentiation of a p53-SLP vaccine by cyclophosphamide in ovarian cancer: A single-arm phase II study. Int. J. Cancer.

[233] Hardwick N.R., Frankel P., Ruel C., Kilpatrick J., Tsai W., Kos F., Kaltcheva T., Leong L., Morgan R., Chung V. (2018). p53-Reactive T-cells Are Associated with Clinical Benefit in Patients with Platinum-Resistant Epithelial Ovarian Cancer After Treatment with a p53 Vaccine and Gemcitabine Chemotherapy. Clin. Cancer Res. Off. J. Am. Assoc. Cancer Res..

[234] Odunsi K., Matsuzaki J., Karbach J., Neumann A., Mhawech-Fauceglia P., Miller A., Beck A., Morrison C.D., Ritter G., Godoy H. (2012). Efficacy of vaccination with recombinant vaccinia and fowlpox vectors expressing NY-ESO-1 antigen in ovarian cancer and melanoma patients. Proc. Natl. Acad. Sci. USA.

[235] Odunsi K., Matsuzaki J., James S.R., Mhawech-Fauceglia P., Tsuji T., Miller A., Zhang W., Akers S.N., Griffiths E.A., Miliotto A. (2014). Epigenetic potentiation of NY-ESO-1 vaccine therapy in human ovarian cancer. Cancer Immunol. Res..

[236] Szender J.B., Papanicolau-Sengos A., Eng K.H., Miliotto A.J., Lugade A.A., Gnjatic S., Matsuzaki J., Morrison C.D., Odunsi K. (2017). NY-ESO-1 expression predicts an aggressive phenotype of ovarian cancer. Gynecol. Oncol..

[237] Sahin U., Derhovanessian E., Miller M., Kloke B.P., Simon P., Löwer M., Bukur V., Tadmor A.D., Luxemburger U., Schrörs B. (2017). Personalized RNA mutanome vaccines mobilize poly-specific therapeutic immunity against cancer. Nature.

[238] Ott P.A., Hu Z., Keskin D.B., Shukla S.A., Sun J., Bozym D.J., Zhang W., Luoma A., Giobbie-Hurder A., Peter L. (2017). An immunogenic personal neoantigen vaccine for patients with melanoma. Nature.

[239] Tran E., Turcotte S., Gros A., Robbins P.F., Lu Y.C., Dudley M.E., Wunderlich J.R., Somerville R.P., Hogan K., Hinrichs C.S. (2014). Cancer immunotherapy based on mutation-specific CD4+ T-cells in a patient with epithelial cancer. Science.

[240] Tanyi J.L., Bobisse S., Ophir E., Tuyaerts S., Roberti A., Genolet R., Baumgartner P., Stevenson B.J., Iseli C., Dangaj D. (2018). Personalized cancer vaccine effectively mobilizes antitumor T-cell immunity in ovarian cancer. Sci. Transl. Med..

[241] Rosenberg S.A., Dudley M.E. (2009). Adoptive cell therapy for the treatment of patients with metastatic melanoma. Curr. Opin. Immunol..

[242] Aoki Y., Takakuwa K., Kodama S., Tanaka K., Takahashi M., Tokunaga A., Takahashi T. (1991). Use of adoptive transfer of tumor-infiltrating lymphocytes alone or in combination with cisplatin-containing chemotherapy in patients with epithelial ovarian cancer. Cancer Res..

[243] Fujita K., Ikarashi H., Takakuwa K., Kodama S., Tokunaga A., Takahashi T., Tanaka K. (1995). Prolonged disease-free period in patients with advanced epithelial ovarian cancer after adoptive transfer of tumor-infiltrating lymphocytes. Clin. Cancer Res. Off. J. Am. Assoc. Cancer Res..

[244] Freedman R.S., Edwards C.L., Kavanagh J.J., Kudelka A.P., Katz R.L., Carrasco C.H., Atkinson E.N., Scott W., Tomasovic B., Templin S. (1994). Intraperitoneal adoptive immunotherapy of ovarian carcinoma with tumor-infiltrating lymphocytes and low-dose recombinant interleukin-2: A pilot trial. J. Immunother. Emphas. Tumor Immunol. Off. J. Soc. Biol. Ther..

[245] Kandalaft L.E., Powell D.J., Chiang C.L., Tanyi J., Kim S., Bosch M., Montone K., Mick R., Levine B.L., Torigian D.A. (2013). Autologous lysate-pulsed dendritic cell vaccination followed by adoptive transfer of vaccine-primed ex vivo co-stimulated T-cells in recurrent ovarian cancer. Oncoimmunology.

[246] Urbanska K., Powell D.J. (2015). Advances and prospects in adoptive cell transfer therapy for ovarian cancer. Immunotherapy.

[247] Tanyi J.L., Stashwick C., Plesa G., Morgan M.A., Porter D., Maus M.V., June C.H. (2017). Possible Compartmental Cytokine Release Syndrome in a Patient With Recurrent Ovarian Cancer After Treatment With Mesothelin-targeted CAR-T Cells. J. Immunother..

[248] Kershaw M.H., Westwood J.A., Parker L.L., Wang G., Eshhar Z., Mavroukakis S.A., White D.E., Wunderlich J.R., Canevari S., Rogers-Freezer L. (2006). A phase I study on adoptive immunotherapy using gene-modified T-cells for ovarian cancer. Clin. Cancer Res. Off. J. Am. Assoc. Cancer Res..

[249] Koneru M., O’Cearbhaill R., Pendharkar S., Spriggs D.R., Brentjens R.J. (2015). A phase I clinical trial of adoptive T-cell therapy using IL-12 secreting MUC-16(ecto) directed chimeric antigen receptors for recurrent ovarian cancer. J. Transl. Med..

[250] Muvarak N.E., Chowdhury K., Xia L., Robert C., Choi E.Y., Cai Y., Bellani M., Zou Y., Singh Z.N., Duong V.H. (2016). Enhancing the Cytotoxic Effects of PARP Inhibitors with DNA Demethylating Agents—A Potential Therapy for Cancer. Cancer Cell.

[251] Chiappinelli K.B., Strissel P.L., Desrichard A., Li H., Henke C., Akman B., Hein A., Rote N.S., Cope L.M., Snyder A. (2015). Inhibiting DNA Methylation Causes an Interferon Response in Cancer via dsRNA Including Endogenous Retroviruses. Cell.

[252] Moufarrij S., Srivastava A., Gomez S., Hadley M., Palmer E., Austin P.T., Chisholm S., Roche K., Yu A., Li J. (2020). Combining DNMT and HDAC6 inhibitors increases antitumor immune signaling and decreases tumor burden in ovarian cancer. Sci. Rep..

[253] Ghoneim H.E., Fan Y., Moustaki A., Abdelsamed H.A., Dash P., Dogra P., Carter R., Awad W., Neale G., Thomas P.G. (2017). De Novo Epigenetic Programs Inhibit PD-1 Blockade-Mediated T-cell Rejuvenation. Cell.

[254] Oza A.M., Matulonis U.A., Alvarez Secord A., Nemunaitis J., Roman L.D., Blagden S.P., Banerjee S., McGuire W.P., Ghamande S., Birrer M.J. (2020). A Randomized Phase II Trial of Epigenetic Priming with Guadecitabine and Carboplatin in Platinum-resistant, Recurrent Ovarian Cancer. Clin. Cancer Res. Off. J. Am. Assoc. Cancer Res..

[255] Fang F., Cardenas H., Huang H., Jiang G., Perkins S.M., Zhang C., Keer H.N., Liu Y., Nephew K.P., Matei D. (2018). Genomic and Epigenomic Signatures in Ovarian Cancer Associated with Resensitization to Platinum Drugs. Cancer Res..

